# Implications of specific lysine residues within ataxin-3 for the molecular pathogenesis of Machado-Joseph disease

**DOI:** 10.3389/fnmol.2023.1133271

**Published:** 2023-05-19

**Authors:** Priscila Pereira Sena, Jonasz Jeremiasz Weber, Sercan Bayezit, Rafael Saup, Rana Dilara Incebacak Eltemur, Xiaoling Li, Ana Velic, Jaqueline Jung, Boris Macek, Huu Phuc Nguyen, Olaf Riess, Thorsten Schmidt

**Affiliations:** ^1^Institute of Medical Genetics and Applied Genomics, University of Tübingen, Tübingen, Germany; ^2^Department of Human Genetics, Ruhr University Bochum, Bochum, Germany; ^3^Proteome Center Tübingen, University of Tübingen, Tübingen, Germany

**Keywords:** Polyglutamine diseases, spinocerebellar ataxia type 3, protein aggregation, proteasomal degradation, deubiquitinase, ataxin-3, posttranslational modification, ubiquitination

## Abstract

Lysine residues are one of the main sites for posttranslational modifications of proteins, and lysine ubiquitination of the Machado-Joseph disease protein ataxin-3 is implicated in its cellular function and polyglutamine expansion-dependent toxicity. Despite previously undertaken efforts, the individual roles of specific lysine residues of the ataxin-3 sequence are not entirely understood and demand further analysis. By retaining single lysine residues of otherwise lysine-free wild-type and polyglutamine-expanded ataxin-3, we assessed the effects of a site-limited modifiability on ataxin-3 protein levels, aggregation propensity, localization, and stability. We confirmed earlier findings that levels of lysine-free ataxin-3 are reduced due to its decreased stability, which led to a diminished load of SDS-insoluble species of its polyglutamine-expanded form. The isolated presence of several single lysine residues within the N-terminus of polyglutamine-expanded ataxin-3 significantly restored its aggregate levels, with highest fold changes induced by the presence of lysine 8 or lysine 85, respectively. Ataxin-3 lacking all lysine residues presented a slightly increased nuclear localization, which was counteracted by the reintroduction of lysine 85, whereas presence of either lysine 8 or lysine 85 led to a significantly higher ataxin-3 stability. Moreover, lysine-free ataxin-3 showed increased toxicity and binding to K48-linked polyubiquitin chains, whereas the reintroduction of lysine 85, located between the ubiquitin-binding sites 1 and 2 of ataxin-3, normalized its binding affinity. Overall, our data highlight the relevance of lysine residues 8 and 85 of ataxin-3 and encourage further analyses, to evaluate the potential of modulating posttranslational modifications of these sites for influencing pathophysiological characteristics of the Machado-Joseph disease protein.

## Introduction

In Machado-Joseph disease (MJD), also known as spinocerebellar ataxia type 3 (SCA3), the affected protein ataxin-3 is characterized by a pathological expansion of a C-terminally located polyglutamine (polyQ) stretch, encoded by a CAG repeat in the *ATXN3* gene. Usually, wild-type ataxin-3 is a predominantly cytoplasmic protein; however, nuclear mislocalization and aggregation of mutant ataxin-3 in neuronal cells are crucial determinants of the disease protein toxicity and the molecular pathogenesis in MJD (Paulson et al., 1997; Schmidt et al., 1998).

Over the last years, posttranslational modifications (PTMs) were demonstrated not only as important regulators of the physiological function of ataxin-3 as a deubiquitinating enzyme (DUB; Burnett et al., 2003), but also of its disease-related features when affected by the polyQ expansion. Multiple studies demonstrated that phosphorylation, proteolytic cleavage, SUMOylation and ubiquitination of ataxin-3 do not only influence protein activity but also strongly modulate the disease-associated properties of its polyQ-expanded form (McLoughlin et al., 2020; Gogia et al., 2022). For instance, phosphorylation or phosphomimetic mutations of S12 or S256 in ataxin-3 showed protective effects, by reducing toxic aggregation of the polyQ-expanded protein *in vitro* or *in vivo* (Fei et al., 2007; Matos et al., 2016), whereas casein kinase 2 (CK2)-associated phosphorylation of ataxin-3 triggered a detrimental nuclear localization and aggregation (Mueller et al., 2009). Proteolysis of ataxin-3 by calpains at amino acids D208 and S256 generated toxic and aggregation-prone C-terminal fragments, and calpain cleavage-resistant ataxin-3 featuring five-amino acid deletions including I253 or G273 reduced fragmentation and aggregation *in vivo* (Weber et al., 2017; Simões et al., 2022). Furthermore, SUMOylation at K166 stabilized polyQ-expanded ataxin-3 and increased its toxicity without changing its localization or aggregation-propensity (Zhou et al., 2013). On the other hand, K356 SUMOylation reduced the formation of ataxin-3 fibrils (Almeida et al., 2015). Ubiquitination of ataxin-3 occurs at numerous sites along the entire protein sequence such as on lysine residues K8, K85, K117, K190, K200, and K291, with K117 and K200 being the primary targets. The distribution of ubiquitination varies between wild-type and polyQ-expanded ataxin-3 (Todi et al., 2010; Kristensen et al., 2017). Among these, the best studied modification is ubiquitination at K117, which was demonstrated to not only increase the DUB activity of ataxin-3 - most likely attributed to its localization within the active site of the enzyme - but also to protect against polyQ-dependent degenerative effects in flies (Todi et al., 2010; Tsou et al., 2013).

As lysine residues are one of the main target sites for PTMs within a protein and some potentially modified sites of ataxin-3 remain incompletely analyzed, we aimed at investigating single lysine residues regarding their contribution to aggregation, localization, stability, degradation, and toxicity of the MJD disease protein. More specifically, we analyzed lysine-free (K0) ataxin-3 as well as single-lysine ataxin-3 variants. The selection of specific sites was done based on the following previously available information: lysine 8 is reported to be ubiquitinated in polyQ-expanded ataxin-3 only (Kristensen et al., 2017); lysine 85 is located inside of one of the nuclear export signals and between ubiquitin binding sites 1 and 2 of ataxin-3 (Blount et al., 2014); lysine 117 is described as the primary site of ubiquitination within ataxin-3 (Todi et al., 2010); lysine 166 is modified by SUMOylation, contributing to cytotoxicity of polyQ-expanded ataxin-3 (Zhou et al., 2013); and lysine 200 is reportedly ubiquitinated in non-pathogenic ataxin-3 only (Todi et al., 2010).

## Materials and methods

### Expression constructs

N-terminally GFP-tagged ataxin-3 (GFP-Atx3) was overexpressed using pEGFP-C2 (Clontech, Mountain View, CA, US) vectors, encoding the canonical isoform 2 (UniProt: P54252-2), also known as ataxin-3c (Goto et al., 1997; Weishäupl et al., 2019), of ataxin-3 featuring three ubiquitin-interacting motifs (UIMs) with 15 or 70 glutamines (15Q or 70Q, respectively). Lysine-free (K0) and single-lysine variants (K8, K85, K117, K166, and K200) of ataxin-3 were generated by a combination of gBlocks™ gene fragments (Integrated DNA Technologies, Inc., Leuven, Belgium) and degenerated primers, designed with Primer 3^1^ and containing sequences for specific restriction sites within ATXN3. All gBlocks™ sequences and a complete list of degenerated primer sequence and employed restriction enzymes are provided in Supplementary Tables S1, S2, respectively. Correct integration of mutations was verified using Sanger sequencing with following primers: forward 1: 5′ - CAGGTTATAAGCAATGCCTTG - 3′; reverse 1: 5′ - GCAATCTGGCAGATCACCC - 3′; forward 2: 5′ - CTCCTGCAGATGATTAGGGT - 3′; forward 3: 5′ - GCTAAGTATGCAAGGTAGTTCC - 3′; reverse 2: 5′ - CAAGTGCTCCTGAACTGGTG - 3′. FLAG-tagged ubiquitin (FLAG-Ub) constructs were a gift from Dr. Guido Krebiehl (former affiliation: German Center for Neurodegenerative Diseases (DZNE), Tübingen, Germany).

### Cell culture

HEK 293 T ataxin-3 knockout (293 T *ATXN3*^−/−^) cells (Weishäupl et al., 2019) were cultured in Dulbecco’s modified Eagle’s medium (DMEM), high glucose, GlutaMAX™ supplemented with 10% (v/v) fetal bovine serum (FBS), 1% (v/v) non-essential amino acids (MEM NEAA) and 1% (v/v) Antibiotic-Antimycotic (A/A) (all Gibco^®^, Thermo Fisher Scientific, Karlsruhe, Germany) and maintained at 5% CO_2_ and 37°C. Transfection with overexpression constructs was conducted using Attractene Transfection Reagent (QIAGEN, Hilden, Germany) or Turbofectin 8.0 (OriGene Technologies, Inc., Rockville, MD, US) according to the manufacturers’ protocols.

### Cycloheximide chase assay

For evaluating ataxin-3 stability, 293 T *ATXN3*^−/−^ cells were seeded in 6-well cell culture plates, transfected 24 h later, and cultured for another 24 h prior to treatment with cycloheximide (CHX; Sigma-Aldrich Chemie, Munich, Germany) at 25 μg/mL for 12 h, 24 h and 36 h. Dimethyl sulfoxide (DMSO; Sigma-Aldrich)-treated cells were used as reference.

### Inhibition of protein degradation

For blocking proteasomal or autophagosomal degradation pathways, cells were treated with 2.5 μM proteasome inhibitor MG132 (Merck) or 25 nM vacuolar-type H + -ATPase inhibitor bafilomycin A1 (BafA1; Enzo Life Sciences, Lörrach, Germany) for 24 h. Before administration, all compounds were prediluted in Opti-MEM^®^ I Reduced Serum Media (Gibco^®^, Thermo Fisher Scientific) and added dropwise to the cell medium. DMSO served as vehicle control.

### Cell viability assay

293T *ATXN3*^−/−^ cells were seeded in 96-well cell culture plates (ViewPlate-96 Black, Perkin Elmer, Massachusetts, USA), transfected 24 h after, and cultured for further 72 h prior to cell viability assessment using PrestoBlue assay (Thermo Fisher Scientific, Karlsruhe, Germany), according to the manufacturer’s protocol. For this, culture medium was replaced by fresh medium containing PrestoBlue reagent in a 1:10 ratio and cells were kept under standard culture conditions. Fluorescence signals were measured 60 min later, using a Synergy HT plate reader and the software Gen5 (both Biotek, Vermont, USA).

### Fluorescence microscopy

For fluorescence microscopy of cells expressing GFP-Atx3, 293 T *ATXN3*^−/−^ cells were plated on poly-L-lysine-coated 8-well Nunc Lab-Tek Chamber Slides (Thermo Fisher Scientific), transfected with respective constructs 24 h after, and cultured for further 72 h. Subsequently, cells were pre-fixed by supplementing the medium with 0.4% (w/v) paraformaldehyde (PFA) and incubating at 37°C for 10 min. Afterwards, medium was aspirated, cells were washed once with 1× DPBS (Gibco^®^, Thermo Fisher Scientific) and incubated in 4% (w/v) PFA in 1× DPBS for 15 min. Next, the fixative was removed, and cells were washed three times with 1× DPBS for 5 min. Finally, cells were mounted with VECTASHIELD Antifade Mounting Medium with DAPI (Vector Laboratories, Peterborough, UK) using coverslips and sealed with transparent nail polish.

Fluorescent images were taken at a 400× magnification on an Axioplan 2 imaging microscope equipped with an ApoTome, a Plan-Neofluar 40×/0.75 objective, and an AxioCam MRm camera, using the AxioVision 4.3 imaging software (all Zeiss, Oberkochen, Germany).

### Protein extraction

For protein extraction, cells were dissociated by gentle pipetting in culture medium and transferred to 2 mL tubes. Cell pellets were obtained by centrifugation at 500 × *g* for 5 min followed by aspiration of the supernatant. Pellets were washed once with cold 1× DPBS. Cell lysis was conducted by resuspending the cell pellet in DPBS-N (1× DPBS with 0.1% (v/v) NP-40) for protein homogenates or RIPA buffer (50 mM Tris pH 7.5, 150 mM NaCl, 10% (v/v) glycerol, 0.1% (w/v) SDS, 0.5% (w/v) sodium deoxycholate and 1% (v/v) Triton X-100) for protein lysates, with both buffers containing cOmplete™ protease inhibitor cocktail and PhosSTOP™ phosphatase inhibitor cocktail (both Roche, Basel, Switzerland), and incubated on ice for 15 min. For cell homogenates, samples were dispersed by ultrasonication using a Sonopuls ultrasonic homogenizer (Bandelin electronic, Berlin, Germany) for 3 s and 10% pulse duration at 10% power. For generating cell lysates, samples were centrifuged at 4°C and 16,100 × *g* for 15 min, and supernatants were transferred to a fresh, pre-cooled reaction tube. Protein concentrations were measured spectrophotometrically in a microtiter plate using Bradford assay reagent (Bio-Rad Laboratories, Basel, Switzerland). Samples were stored at −80°C until further analysis.

### Immunoprecipitation

FLAG- and GFP-tagged proteins were immunoprecipitated using DYKDDDDK Fab-Trap and GFP-Trap agarose, both according to the manufacturer’s protocol (ChromoTek, Planegg-Martinsried, Germany) with the following modifications: Transfected cells were harvested by resuspension in culture medium (content of two wells of a 6-well plate) and subsequent centrifugation for 5 min at 4°C and 300 × *g*. Cell pellets were washed once with 1× DPBS and centrifuged again. Afterwards, cell pellets were lysed in 150 μL Trap lysis buffer (10 mM Tris pH 7.5, 150 mM NaCl, 0.5 mM EDTA, 0.5% IGEPAL CA-630) containing EDTA-free cOmplete protease inhibitor cocktail and PhosSTOP phosphatase inhibitor cocktail (both Roche). After measuring protein concentrations using Bradford reagent, lysate concentrations were adjusted with Trap lysis buffer to 5 μg/μL and further diluted in Trap dilution buffer (10 mM Tris pH 7.5, 150 mM NaCl, 0.5 mM EDTA) with protease and phosphatase inhibitors to a final concentration of 2 μg/μL. For immunoprecipitation, 500 μg total protein were incubated with 10 μL of agarose bead slurry in a 1.5 mL reaction tube, rotating end-over-end at 4°C and 12 rpm for 1 h. Subsequently, beads were washed with Trap wash buffer (10 mM Tris pH 7.5, 150 mM NaCl, 0.5 mM EDTA, 0.05% IGEPAL CA-630) containing EDTA-free protease and phosphatase inhibitors, and heat-denatured at 70°C for 10 min in 90 μL of 4× LDS sample buffer (1 M Tris pH 8.5, 50% (v/v) glycerol, 8% (w/v) LDS, 2 mM EDTA, 0.1% (w/v) Orange G) mixed with Trap dilution buffer in a ratio 1:1 and supplemented with 100 mM dithiothreitol (DTT). Samples were subsequently analyzed by western blotting.

### NanoLC-MS/MS analysis and data processing

Immunoprecipitated GFP-tagged ataxin-3 variants were submitted to a short-run SDS-PAGE electrophoresis, followed by gel fixation and staining using colloidal Coomassie brilliant blue (CBB-G250) staining solution (0.02% CBB-G250, 2% (w/v) phosphoric acid, 5% aluminum sulfate, 10% ethanol), according to Kang et al. (2002). Afterwards, gel was de-stained with a 10% ethanol and 2% ortho-phosphoric acid and rinsed with ultrapure water. Gel lanes containing samples were excised and in-gel digested using trypsin as described previously (Borchert et al., 2010). After desalting using C18 Stage tips (Rappsilber et al., 2007) extracted peptides were separated on an Easy-nLC 1200 system coupled to a Q Exactive HF mass spectrometer (both Thermo Fisher Scientific) as described elsewhere (Kliza et al., 2017) with small modifications: The peptide mixtures were separated using a 1-h gradient and the seven most intense precursor ions were sequentially fragmented in each scan cycle using higher energy collisional dissociation (HCD) fragmentation.

Acquired MS spectra were processed with MaxQuant software package version 1.6.7.0 (Cox and Mann, 2008) with integrated Andromeda search engine (Cox et al., 2011). Database search was performed against a target-decoy *Homo sapiens* database obtained from UniProt, containing 96,817 protein entries and 286 commonly observed contaminants. Additionally, one small database was created containing sequences used for pulldown. Endoprotease trypsin was defined as protease with a maximum of two missed cleavages. Oxidation of methionine and N-terminal acetylation were specified as variable modifications, whereas carbamidomethylation on cysteine was set as fixed modification. Search for GlyGly(K) modification was enabled. Initial maximum allowed mass tolerance was set to 4.5 parts per million (ppm) for precursor ions and 20 ppm for fragment ions. Peptide, protein and modification site identifications were reported at a false discovery rate (FDR) of 0.01, estimated by the target/decoy approach (Elias and Gygi, 2007). Data was filtered for contaminants, reverse and only identified by site entries.

### Subcellular fractionation assay

Investigation of the subcellular localization of ataxin-3 was performed following the Rapid, Efficient And Practical (REAP) nucleocytoplasmic fractionation protocol (Suzuki et al., 2010) with small modifications (Weber et al., 2017; Abeditashi et al., 2022). 293 T *ATXN3*^−/−^ cells were harvested 72 h post-transfection using DPBS and centrifuged at 300 × *g* for 5 min. Cell pellets were triturated in DPBS-N buffer supplemented with EDTA-free cOmplete protease inhibitor, and an aliquot of the cell suspension was transferred to a fresh tube representing the whole cell extract. The residual volume was centrifuged at 10,000 × *g* for 10 s and the supernatant was collected as the cytoplasmic fraction. Whole cell extract and cytoplasmic fraction were mixed with 4× LDS sample buffer in a ratio 3:1 and supplemented with 100 mM DTT. The nuclear fraction was obtained by resuspending the pellet in 1× LDS sample buffer containing 100 mM DTT. All samples were heat-denatured for 10 min at 70°C followed by ultrasonication for 10 s. Samples were then analyzed via western blotting. Ataxin-3 signals were normalized to nuclear loading control protein lamin A/C in the whole cell extract and nuclear fraction of each condition. Finally, the percentage of nuclear ataxin-3 in relation to the amount of ataxin-3 in the whole cell extract was calculated.

### SDS-PAGE and western blotting

Western blotting was performed according to standard procedures. Briefly, protein from cell homogenates or lysates were mixed with a 4× LDS sample buffer in a ratio 3:1 and supplemented with 100 mM DTT. After heat-denaturing for 10 min at 70°C, protein samples were electrophoretically separated on custom-made Bis-Tris polyacrylamide gels and MOPS running buffer (50 mM MOPS, 50 mM Tris pH 7.7, 0.1% (w/v) SDS, 1 mM EDTA) using a Mini-Protean Tetra Cell electrophoresis system (Bio-Rad Laboratories) or on pre-cast 3–8% NuPAGE^®^ Tris-Acetate gradient gels (Thermo Fisher Scientific) and Tris-acetate running buffer (50 mM Tricine, 50 mM Tris pH 8.25, 0.1% (w/v) SDS) using a Mini Gel Tank (Thermo Fisher Scientific). Proteins were transferred on Amersham™ Protran™ Premium 0.2 μm nitrocellulose membranes (Cytiva, Freiburg, Germany) using Bicine/Bis-Tris transfer buffer (25 mM Bicine, 25 mM Bis-Tris pH 7.2, 1 mM EDTA, 15% (v/v) methanol) and a TE22 Transfer Tank (Hoefer, Inc., Holliston, MA, US) at 80 V and a maximum of 250 mA for 1.5–2.0 h. Where necessary, total protein staining was performed using SYPRO Ruby blot stain (Thermo Fisher Scientific) following the manufacturer’s instructions. Afterwards, membranes were blocked with 5% (w/v) skim milk powder in 1× TBS (10 mM Tris pH 7.5, 150 mM NaCl) at room temperature for 1 h, and probed with primary antibodies diluted in 1× TBS-T (1× TBS with 0.1% (v/v) Tween 20) at 4°C overnight. A detailed listing of applied primary antibodies can be found in Supplementary Table S3. Subsequently, membranes were washed with 1× TBS-T and incubated with the respective secondary IRDye^®^ antibodies goat anti-mouse 680LT, goat anti-mouse 800CW, or goat anti-rabbit 800CW (all 1:5000; LI-COR Biosciences, Lincoln, NE, US). After final washing with 1× TBS-T, fluorescence signals were detected using the LI-COR ODYSSEY^®^ FC and quantified with Image Studio 4.0 software (both LI-COR Biosciences).

### Filter retardation assay

Detection of SDS-insoluble ataxin-3 species was performed as previously described (Pereira Sena et al., 2021). In brief, 3–6 μg of protein from cell homogenates were diluted in 1× DPBS containing 2% (w/v) SDS and 50 mM DTT. Then, samples were heat-denatured at 95°C for 5 min and filtered through a nitrocellulose membrane (0.45 μm, Cytiva) using a Minifold^®^ II Slot Blot System (Schleicher & Schüll, Dassel, Germany). Membranes were incubated with the mouse anti-GFP primary antibody (1:1000, sc-9996, Santa Cruz Biotechnology, Inc., Dallas, TX, US) at 4°C overnight and goat anti-mouse 800CW secondary antibody (1:5000, LI-COR Biosciences) at room temperature for 1 h. Fluorescence signals were detected using the LI-COR ODYSSEY FC and quantified with Image Studio 4.0 software (both LI-COR Biosciences).

### RNA extraction, cDNA synthesis, and quantitative real-time PCR

For analysis of mRNA expression using quantitative real-time PCR (qRT-PCR), RNA was extracted from cell culture samples using the RNeasy Mini Kit (QIAGEN) according to the manufacturer’s protocol. Cells were harvested as described before and lysed using QIAshredder columns. DNA contaminations were removed by employing the RNAse-free DNAse set (both QIAGEN). For reverse transcription, 1 μg of RNA were transcribed to cDNA using the QuantiTect Reverse Transcription Kit (QIAGEN) according to the manufacturer’s protocol. GFP-tagged *ATXN3* mRNA expression was assessed using the following primers for GFP: fwd 5′ - CCTCACGTATGGCGTTCAGT - 3′, rev 5′ - GTTCCTGGACGTAGCCTTCC - 3′. Expression of the housekeeping genes *GAPDH* (fwd 5′ - GCTCTCTGCTCCTCCTGTTC - 3′, rev 5′ - ACGACCAAATCCGTTGACTC - 3′) and *SDHA* (fwd 5′ - AGAAGCCCTTTGAGGAGCA - 3′, rev 5′ - CGATTACGGGTCTATATTCCAGA - 3′) was used as a reference for normalization. qRT-PCR was performed using the QuantiTect SYBR Green PCR-Kit (QIAGEN) in a LightCycler 480 (Roche Diagnostics, Mannheim, Germany). Relative expression levels of GFP-tagged *ATXN3* mRNA were calculated based on the Pfaffl (2001) model after normalization to the reference genes.

### Statistical analyses

Data were analyzed using GraphPad Prism 9.1.2 (GraphPad Software, San Diego, CA, US). Results are presented as individual data points with means ± SEM. One-sample *t*-test, Student’s *t*-test, or one-way ANOVA with Dunnett or Tukey *post hoc* test were applied. Statistical significance was assumed with a *value of p* ≤0.05. For further details, see respective figure legends.

## Results

### Protein levels of lysine-free ataxin-3 are reduced despite unchanged mRNA expression

**Figure 1:**
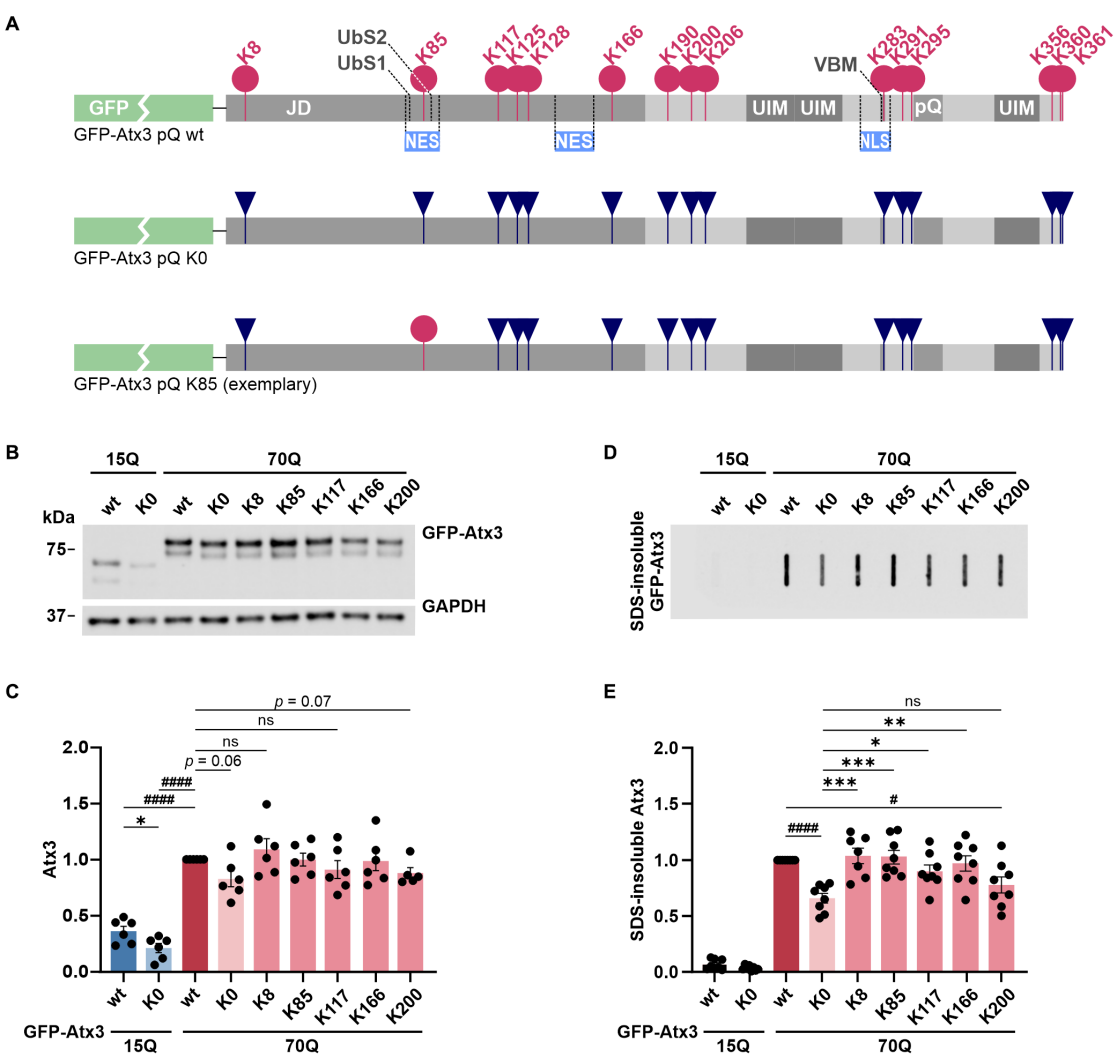
Lysine-free ataxin-3 shows reduced soluble and SDS-insoluble protein levels, while specific single lysine residues offset this effect. **(A)** Schematic of the construct design for ataxin-3 used in this study. All lysine residues (K; pink circles) within the N-terminally GFP-tagged Atx3 (GFP-Atx3) were converted to arginine (R; dark blue inverted triangles). Single lysine residues of interest were reintroduced (exemplified by a pink circle for K85). JD, Josephin domain; UbS, ubiquitin-binding site; NES, nuclear export signal; UIM, ubiquitin-interacting motif; VBM, VCP-binding motif; NLS, nuclear localization signal; pQ, polyglutamine stretch. **(B)** Western blot analysis of protein extracts from ataxin-3 knockout HEK 293 T cells (293 T *ATXN3*^−/−^) transfected with GFP-Atx3 variants. GAPDH served as loading control. **(C)** Densitometric quantification of soluble ataxin-3 protein, normalized to GAPDH and relative to Atx3 70Q wt, demonstrated reduced levels of Atx3 15Q (wt and K0), 70Q K0 and 70Q K200 *n* = 6. 15Q wt and 15Q K0 vs. 70Q wt, both *p* < 0.0001 (#, one-sample *t*-test); 15Q wt vs. 15Q K0, *p* = 0.03 (*, unpaired *t*-test); ns = not significant. **(D)** Filter retardation assay for analysis of SDS-insoluble forms of ataxin-3 in the same protein extracts. **(E)** Densitometric quantification of SDS-insoluble ataxin-3 demonstrated a reduction in aggregate level of Atx3 70Q K0 and 70Q K200 in comparison to 70Q wt. *n* = 6. 70Q wt vs. 70Q K0, *p* = 0.0001 and 70Q wt vs. 70Q K200, *p* = 0.02 (#, one-sample *t*-test); 70Q K0 vs. 70Q K8, *p* = 0.0007; vs. 70Q K85, *p* = 0.0007; vs. 70Q K117, *p* = 0.04; vs. 70Q K166, *p* = 0.004 (*, ordinary one-way ANOVA with Dunnett *post hoc* test). Data is represented as means ± SEM. *, ^#^ *p* ≤ 0.05, **, ^##^ *p* ≤ 0.01, ***, ^###^ *p* ≤ 0.001, ****, ^####^ *p* ≤ 0.0001.

Within the amino acid sequence of proteins, lysine residues represent the main target site for ubiquitination, a PTM primarily associated with protein degradation (Dikic, 2017). To analyze the functional and proteostatic relevance of lysine residues in the first two hundred amino acids of ataxin-3, we generated a set of N-terminally GFP-tagged constructs (GFP-Atx3) in which all lysine within ataxin-3 were mutated to arginine, as well as constructs containing single lysine residues. Furthermore, all constructs of interest were generated in a wild-type (15Q) and a polyQ-expanded form (70Q) to assess the impact on the pathogenicity of ataxin-3. A representation of some of the constructs used in the study is provided in Figure 1A. For a schematic overview of all employed constructs see Supplementary Figure S1.

In a first step, we tested whether all constructs are expressed at equal protein levels in a standard cell model. For this, HEK 293 T ataxin-3 knockout cells (293 T *ATXN3^−/−^*) were transiently transfected with constructs expressing either wild-type GFP-Atx3 with 15 glutamines (15Q wt), polyQ-expanded GFP-Atx3 with 70 glutamines (70Q wt), lysine-free versions of both polyQ expansions (15Q K0 and 70Q K0, respectively) or polyQ-expanded GFP-Atx3 with single lysine residues (70Q K8, 70Q K85, 70Q K117, 70Q K166, or 70Q K200), and analyzed by western blotting. Interestingly, we detected a strong difference between Atx3 15Q and 70Q, with the 70Q form exhibiting about 150% higher protein levels (Figures 1B,C). Moreover, lysine-free ataxin-3 (K0) showed significantly lower protein levels for its 15Q form as well as a strong trend toward reduction for Atx3 70Q when compared to the unmutated counterparts. The presence of single lysine residues K8, K85, K117, or K166 recovered the protein levels in comparison to baseline, while Atx3 K200 showed a robust trend toward reduction (Figures 1B,C). To determine whether the differences observed in the protein levels already occurred at the mRNA levels, we analyzed the expression of our ataxin-3 constructs by qRT-PCR. Our results did not reveal any alteration at the mRNA level (Supplementary Figure S2), suggesting posttranslational effects as the root for the higher Atx3 70Q protein amounts compared to 15Q, and for the reduction in Atx3 15Q K0, 70Q K0, and 70Q K200 levels. As ataxin-3 is known to be a substrate for caspase- and calpain-dependent proteolysis (Matos et al., 2016), we tested whether the decreased levels of Atx3 15Q K0, 70Q K0, and 70Q K200 coincide with an increase of potential proteolytic fragments. Western blot analysis using antibodies against the GFP-tag or an epitope at the N-terminus of ataxin-3 did detect specific cleaved fragments. However, these fragments did not increase in response to the presence or absence of lysine residues, contradicting a direct effect of lysine substitution on cleavage sites and proteolysis by enzymes such as caspases or calpains (Supplementary Figure S3).

### Lysine residues K8, K85, K117, and K166 uphold levels of ataxin-3 aggregation

A central pathological hallmark of MJD is the aggregation propensity of polyQ-expanded ataxin-3, which accumulates in the nucleus and axons of neuronal cells as inclusion bodies, ultimately leading to their degeneration (Paulson et al., 1997; Schmidt et al., 1998; Seidel et al., 2010). As the observed variation in soluble levels of our ataxin-3 lysine-free and single-lysine constructs might be connected to changes in its aggregation load, we investigated whether the absence of all or presence of individual lysine residues may influence the aggregation behavior of polyQ-expanded ataxin-3.

Since our GFP-Atx3 70Q constructs do not undergo a microscopically detectable inclusion body formation when compared to hyperexpanded constructs containing 148Q or more (Supplementary Figure S4), we analyzed smaller, SDS-insoluble forms of aggregated ataxin-3 using filter retardation assays. Analysis of protein homogenates of 293 T *ATXN3*^−/−^ cells overexpressing different GFP-Atx3 constructs for 96 h using a GFP tag-specific antibody demonstrated a strong abundance of SDS-insoluble GFP-Atx3 70Q when compared to the 15Q wild-type counterpart (Figure 1D).

Interestingly, the load of SDS-insoluble forms of the Atx3 70Q K0 variant was strongly reduced, even to a greater extent than the soluble levels of the protein. Presence of lysine residues K8, K85, K117, or K166 counteracted this drop in insoluble ataxin-3 species, whereas K200 resembled the K0 condition (Figure 1D). Densitometric analysis of the filter retardation assays demonstrated that lysine residues K8 and K85 had the strongest effect on preventing the drop in ataxin-3 aggregation (Figure 1E).

### Lysine-free ataxin-3 shows an enriched nuclear localization, whereas K85 restores an even nucleocytoplasmic distribution of ataxin-3

In previous studies, we demonstrated that the subcellular localization of ataxin-3 can be modulated by mutating amino acids in its localization signals, or by the overexpression of nucleocytoplasmic transport proteins with consequences on the aggregation behavior (Antony et al., 2009; Sowa et al., 2018). Specifically, nuclear localization of ataxin-3 worsens the formation of protein aggregates (Bichelmeier et al., 2007). Moreover, the N-terminus of ataxin-3 was shown to be a determinant of the nucleocytoplasmic transport of ataxin-3, and two nuclear export signals (NES), namely NES 77 (I77-L89) and NES 141 (E141-E158), were identified within its Josephin domain, suggesting a cytoplasmic retaining effect on ataxin-3 which might influence intranuclear inclusion body formation (Antony et al., 2009; Macedo-Ribeiro et al., 2009). Importantly, a forced nuclear export by addition of NES to polyQ-expanded ataxin-3 was sufficient to drastically lower intranuclear inclusion body formation *in vivo* (Bichelmeier et al., 2007).

**Figure 2:**
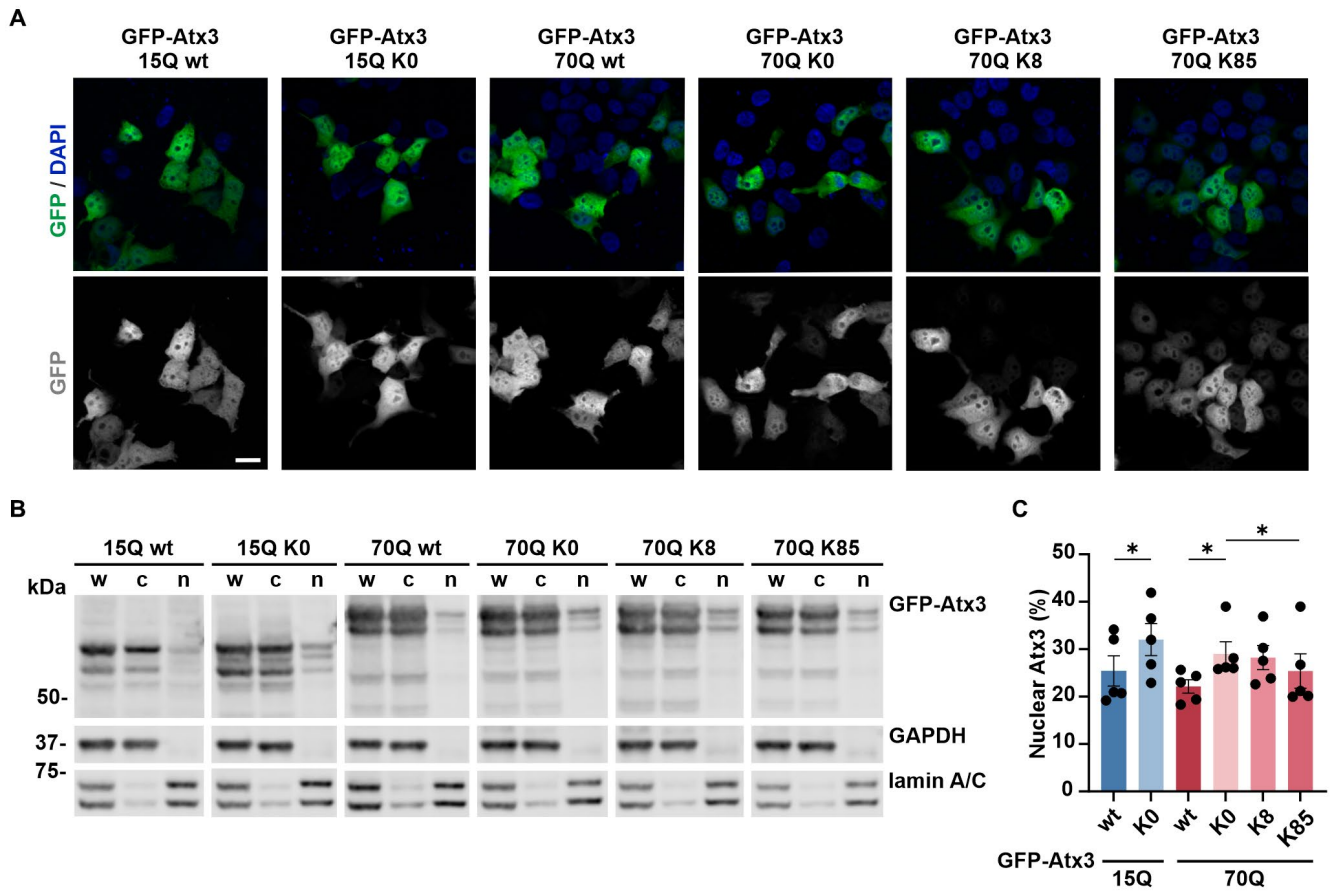
Lysine residues impact the intracellular distribution of ataxin-3. **(A)** Fluorescence microscopy analysis demonstrated a comparable intracellular distribution of the various GFP-Atx3 species, excepting for an apparently stronger nuclear localization of GFP-Atx3 15Q K0 and 70Q K0 in transfected 293 T *ATXN3*^−/−^ cells. Scale bar = 20 μm. **(B)** Western blot analysis of a subcellular fractionation of 293 T *ATXN3*^−/−^ cells overexpressing wild-type, lysine-free and single-lysine ataxin-3 constructs confirmed the trend of an enriched nuclear localization of soluble lysine-free ataxin-3 (15Q K0 and 70Q K0) and demonstrated a rescue of the subcellular distribution with the presence of K85. GAPDH and lamin A/C served as enrichment markers for the cytoplasmic and nuclear fractionation (respectively), and as loading controls. w, whole cell homogenate; c, cytoplasmic fraction; *n*, nuclear fraction. **(C)** Densitometric quantification of the nuclear fraction of ataxin-3 from the experiments represented in **(B)**. Percentages were calculated relative to ataxin-3 in the respective whole cell extract, after normalization to the nuclear protein lamin A/C. *n* = 5, 15Q wt vs. 15Q K0, *p* = 0.02; 70Q wt vs. 70Q K0, *p* = 0.03; 70Q K0 vs. 70Q K85, *p* = 0.01 (*, paired *t*-test). Data is represented as means ± SEM. * *p* ≤ 0.05.

Based on these findings, we sought to investigate whether lysine-free or single-lysine ataxin-3 shows an altered intracellular distribution which might explain differences observed on both protein and aggregation load. To evaluate potential changes in the localization of our ataxin-3 variants, we overexpressed GFP-Atx3 constructs in 293 T *ATXN3*^−/−^ cells and performed analysis by fluorescence microscopy at 72 h post transfection. The visual comparison of the cellular distribution of GFP-Atx3 15Q or 70Q with their respective K0 variants pointed to a slightly increased nuclear localization of the latter, whereas 70Q constructs carrying single lysine residues at positions K8 or K85 did not show a clear trend toward ataxin-3 compartmentalization (Figure 2A). We thus performed a subcellular fractionation analysis under the same experimental conditions, for a quantitative assessment of ataxin-3 subcellular distribution. Western blot analysis of the cytoplasmic and nuclear fractions demonstrated a stronger nuclear localization of lysine-free ataxin-3 regardless of its polyQ length (Figures 2B,C). Interestingly, lysine residue K85 restored the intracellular distribution of Atx3 70Q, whereas Atx3 70Q K8 showed an enrichment in the nuclear fraction similar to the K0 variant.

In conclusion, single lysine residue K85 was able to restore the subcellular distribution of ataxin-3 in comparison to lysine-free ataxin-3, which showed a more nuclear localization than the unmutated form of the protein.

### Stability of ataxin-3 is increased by both K8 and K85

**Figure 3:**
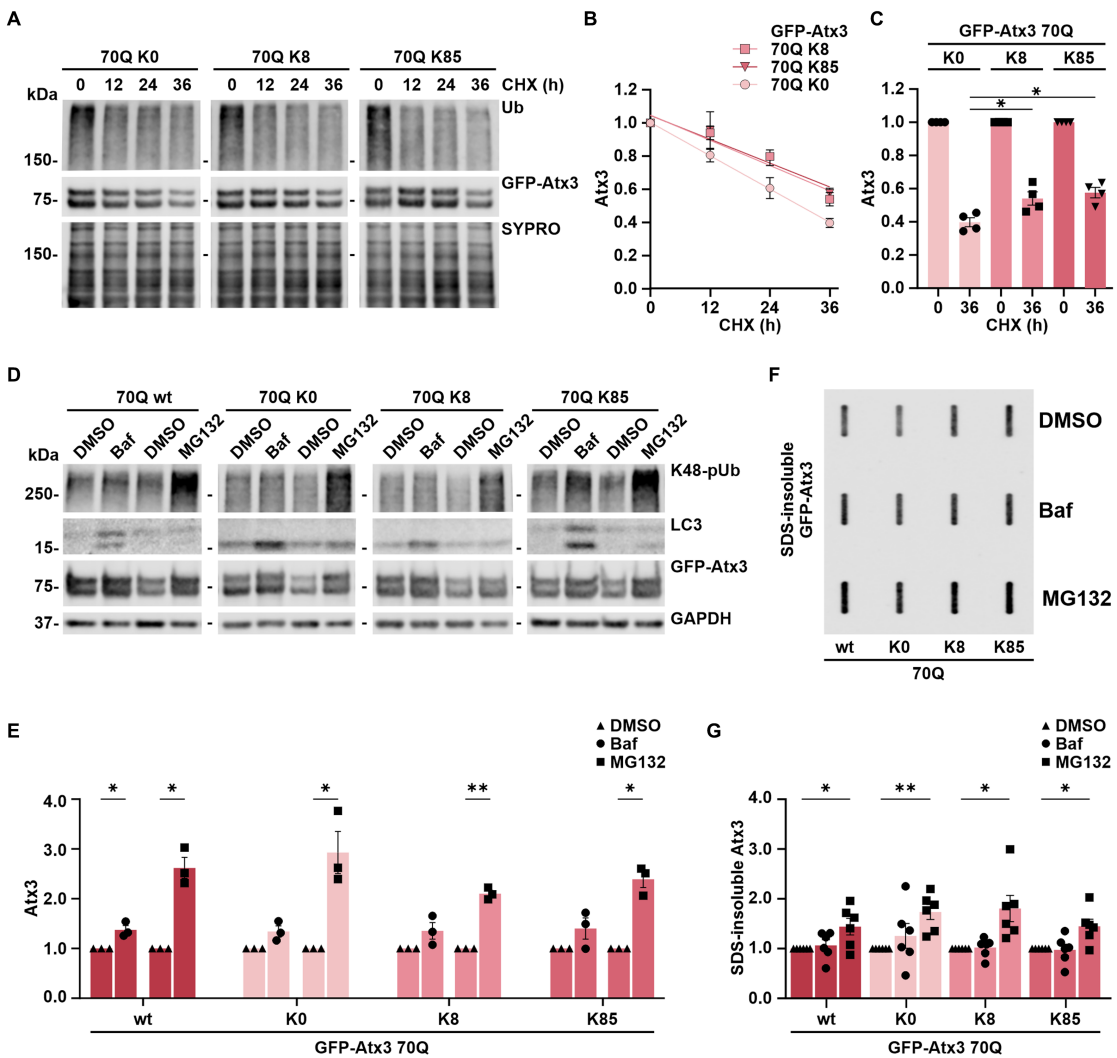
Stability of ataxin-3 is increased by the presence of lysine residues 8 and 85, and ataxin-3 turnover is largely depending on proteasomal degradation. **(A)** Time-dependent reduction of ubiquitin (Ub) chains and degradation of GFP-Atx3 in cells treated with cycloheximide (CHX) for assessing the degradation rate of the different ataxin-3 variants. SYPRO Ruby staining served as loading control. **(B)** Densitometric quantification of soluble ataxin-3 protein from **(A)**, normalized to SYPRO and relative to the timepoint 0 h, demonstrated an increased stability of Atx3 70Q K8 and K85 in comparison to K0. *n* = 4, 70Q K0 vs. 70Q K8 and vs. 70Q K85, *p* = 0.009 (reject null hypothesis that one curve fits all datasets) (One phase decay). **(C)** Densitometric quantification of soluble ataxin-3 protein depicting the CHX chase assay’s endpoint (36 h). *n* = 4, 70Q K0 vs. 70Q K8, *p* = 0.03; vs. 70Q K85, *p* = 0.01 (*, one-way ANOVA with Tukey post-hoc test). **(D)** Western blot of lysates from cells overexpressing the ataxin-3 variants and incubated with either DMSO, autophagy inhibitor bafilomycin (Baf) or the proteasome inhibitor MG132 for 24 h prior to harvesting. Autophagy inhibition led to accumulation of LC3, and the proteasome inhibition resulted in increased K48-linked polyubiquitin chains (K48-pUb). GAPDH served as loading control. **(E)** Densitometric quantification of soluble ataxin-3, normalized to GAPDH and relative to the respective DMSO control. Significantly increased GFP-Atx3 70Q wt levels were observed upon inhibition of both degradation pathways, whereas a statistically significant accumulation of 70Q K0, 70Q K8 and 70Q K85 was only observed upon proteasome inhibition. *n* = 3, 70Q wt Baf vs. DMSO, *p* = 0.05; 70Q wt MG132 vs. DMSO, *p* = 0.02; 70Q K0 MG132 vs. DMSO, *p* = 0.05; 70Q K8 MG132 vs. DMSO, *p* = 0.004; 70Q K85 MG132 vs. DMSO, *p* = 0.01 (*, one-sample *t*-test). **(F)** Accumulation of ataxin-3 species due to the inhibition of degradation pathways had a direct impact on aggregate load, as demonstrated on the filter retardation analysis. **(G)** Densitometric quantification of SDS-insoluble ataxin-3 from **(F)** demonstrated its significant accumulation upon proteasome inhibition in comparison to DMSO control. *n* = 6, 70Q wt MG132 vs. DMSO, *p* = 0.05; 70Q K0 MG132 vs. DMSO, *p* = 0.005; 70Q K8 MG132 vs. DMSO, *p* = 0.03; 70Q K85 MG132 vs. DMSO, *p* = 0.03 (*, one-sample *t*-test). Data is represented as means ± SEM. * *p* ≤ 0.05, ** *p* ≤ 0.01.

As we could not conclusively relate the K8 and K85-linked levels of soluble and aggregated Atx3 70Q with the intracellular distribution of the protein (Figure 2), we sought to investigate whether these lysine residues had any influence on ataxin-3 protein stability. For this, we performed cycloheximide (CHX) chase assays with 293 T *ATXN3*^−/−^ cells overexpressing GFP-Atx3 70Q K0, 70Q K8, or 70Q K85 for up to 36 h and analyzed the stability of the proteins via western blotting. We measured total ubiquitin (Ub) levels as positive control for the assay efficacy (Figure 3A). Compared to Atx3 70Q K0, both the K8 and K85 forms presented a higher stability (Figure 3B, Supplementary Figure S5), suggesting a major role of these residues in ataxin-3 proteostasis. The strongest difference in protein stability of the two single-lysine ataxin-3 forms in comparison to lysine-free ataxin-3 occurred at the 36-h endpoint of the assay (Figures 3B,C).

This assay, therefore, demonstrated that ataxin-3 acquires increased stability upon the presence of lysine residues K8 or K85.

### Ataxin-3 is primarily degraded via the proteasome in HEK 293T cells

The autophagy pathway, together with the ubiquitin-proteasome system (UPS), bear the prime responsibility for degrading most of the intracellular proteins and thereby contribute to a functional proteostasis (Lilienbaum, 2013). Degradation of ataxin-3 has been associated with both proteolytic machineries, with some studies hinting toward isoform-dependent differences in the breakdown of the protein as well as ubiquitination-independent mechanisms (Jana et al., 2005; Harris et al., 2010; Menzies et al., 2010; Blount et al., 2014; Weishäupl et al., 2019; Pereira Sena et al., 2021). Since Atx3 70Q K8 and K85 presented an increased stability in our cycloheximide chase assays compared to the lysine-free variant, we aimed at further elaborating whether single lysine residues may influence the mode of ataxin-3’s degradation via autophagy or the UPS. For this, 293 T *ATXN3*^−/−^ cells overexpressing GFP-Atx3 70Q wt, 70Q K0, 70Q K8 or 70Q K85 were treated for 24 h with either the autophagy inhibitor bafilomycin A1 (BafA1) or the proteasome inhibitor MG132, starting at 24 h post transfection. Protein extracts were then analyzed via western blotting. Accumulation of proteasomal degradation-associated K48-linked polyubiquitin (K48-pUb) chains was used as a marker for proteasomal inhibition, whereas increase of autophagy-related protein LC3 levels was used as a marker for inhibition of autophagy. Our analysis demonstrated that all Atx3 70Q variants, regardless of the presence or absence of all lysine residues or of the presence of single residues K8 or K85, are degraded via both pathways (Figures 3D,E), with proteasomal degradation appearing to be the main responsible for ataxin-3 turnover (Figure 3E).

As Atx3 70Q degradation strongly relied on the UPS, we investigated the consequences of inhibiting autophagy or the UPS on the levels of SDS-insoluble ataxin-3. For this purpose, 293 T *ATXN3*^−/−^ cells overexpressing GFP-Atx3 70Q wt, 70Q K0, 70Q K8 or 70Q K85 were treated for 24 h with BafA1 or MG132 starting at 72 h post transfection. Inhibition of UPS promoted further accumulation of ataxin-3 aggregates, regardless of the lysine variant analyzed, whereas autophagy inhibition did not significantly change the amount of ataxin-3 aggregates (Figures 3F,G). Therefore, we concluded that increased levels of SDS-insoluble ataxin-3 upon MG132 treatment directly reflect the accumulation of soluble ataxin-3 and, thus, proteasomal degradation is the main modifier of the ataxin-3 aggregation process.

### Lysine-free ataxin-3 induces cell death via apoptosis

**Figure 4:**
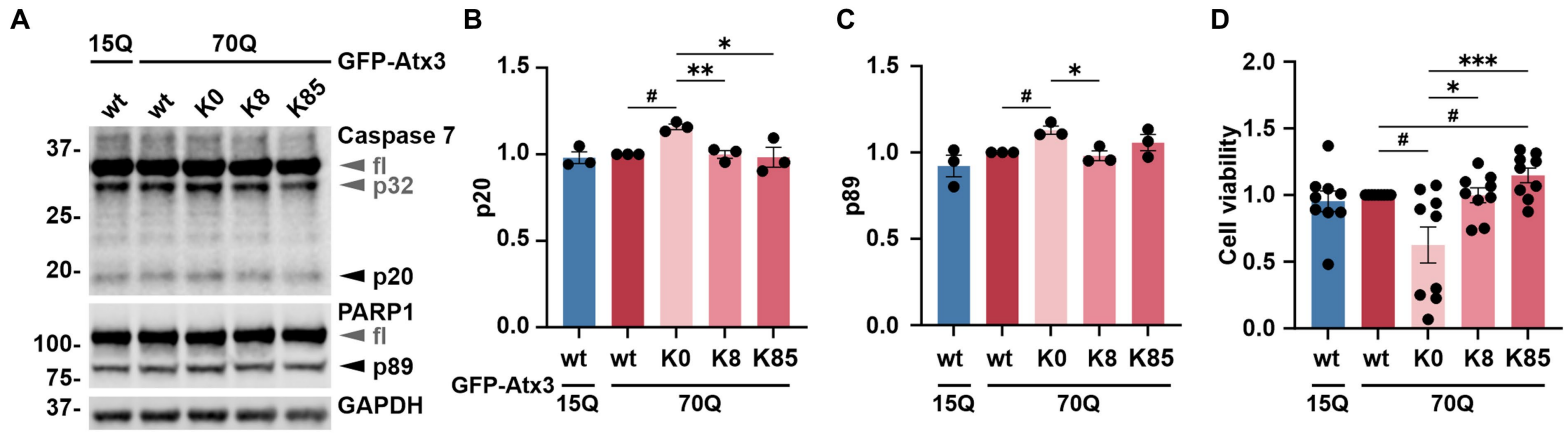
Lysine-free ataxin-3 expression increases apoptotic markers and lowers cell viability. **(A)** Western blot of lysates from cells overexpressing the ataxin-3 variants and incubated with MG132 for 24 h prior to harvesting. Samples expressing Atx3 70Q K0 presented elevated levels of a proteolytic activation of caspase 7 (p32 and p20) and caspase-dependent PARP1 cleavage (p89), suggesting increased apoptosis under this condition. GAPDH served as loading control. fl = full-length. **(B)** Densitometric quantification of the p20 subunit of caspase 7, normalized to full-length caspase 7 and relative to Atx3 70Q wt. *n* = 3, 70Q wt vs. 70Q K0, *p* = 0.01 (#, one-sample *t*-test); 70Q K0 vs. 70Q K8, *p* = 0.005; vs. 70Q K85, *p* = 0.04 (*, unpaired *t*-test). **(C)** Quantification of the p89 fragment of PARP1 normalized to full-length PARP1 and relative to Atx3 70Q wt. n = 3, 70Q wt vs. 70Q K0, *p* = 0.03 (#, one-sample *t*-test); 70Q K0 vs. 70Q K8, *p* = 0.02 (*, unpaired *t*-test). **(D)** Analysis of cell viability relative to Atx3 70Q wt via PrestoBlue assay. GFP-Atx3 constructs were overexpressed in 293 T *ATXN3*^−/−^ cells and treated with MG132 for 24 h prior to the assessment of cell viability. Atx3 70Q K0 lowered the cell viability, whereas the presence of K8 or K85 rescued viability, with K85 showing beneficial effects in comparison to Atx3 70Q wt. *n* = 9, 70Q wt vs. 70Q K0, *p* = 0.02; vs. 70Q K85, *p* = 0.03 (#, one-sample *t*-test); 70Q K0 vs. 70Q K8, *p* = 0.03; vs. 70Q K85, *p* = 0.0006 (*, ordinary one-way ANOVA with Dunnett *post hoc* test). Data is represented as means ± SEM. *, ^#^ *p* ≤ 0.05, **, ^##^ *p* ≤ 0.01, ***, ^###^ *p* ≤ 0.001.

Ataxin-3 is a reported regulator of apoptosis (Liu et al., 2016), a programmed cell death process marked by the proteolytic activation of cysteinyl-aspartate proteases (caspases) and further cleavage of downstream substrates such as the poly [ADP-ribose] polymerase 1 (PARP1) (Chaitanya et al., 2010). Moreover, polyQ-expanded ataxin-3 was shown to exacerbate this physiological process, triggering increased cell death and thereby neurodegeneration (Liu et al., 2016). To evaluate whether the accumulation of ataxin-3 impacted cell apoptosis, we assessed levels of the executioner caspase 7 subunit p20 as well as the PARP1 fragment p89 in 293 T *ATXN3*^−/−^ cells transfected with either GFP-Atx3 15Q wt, 70Q wt, 70Q K0, 70Q K8, or 70Q K85 for 72 h and treated with MG132 for the last 24 h to induce proteotoxic stress. Western blot analysis of cells expressing Atx3 70Q K0 showed a significant increase in both p20 caspase 7 and p89 PARP1 levels in comparison to Atx3 70Q wt, which was not observed for the 70Q K8 and 70Q K85 variants (Figures 4A–C). To evaluate whether the increase in apoptotic markers in Atx3 70Q K0-transfected cells also had consequences on cell viability, we performed the resazurin-based PrestoBlue assay under corresponding conditions. Here, we observed a significant reduction in viability for cells expressing Atx3 70Q K0 in comparison to those expressing Atx3 70Q wt (Figure 4D). Moreover, lysine residues 8 or 85 rescued cell viability. Interestingly, cells transfected with Atx3 70Q K85 had improved viability over cells expressing Atx3 70Q wt (Figure 4D).

### K8 influences ataxin-3 ubiquitination, whereas K85 does not represent a major ubiquitination site within ataxin-3

**Figure 5:**
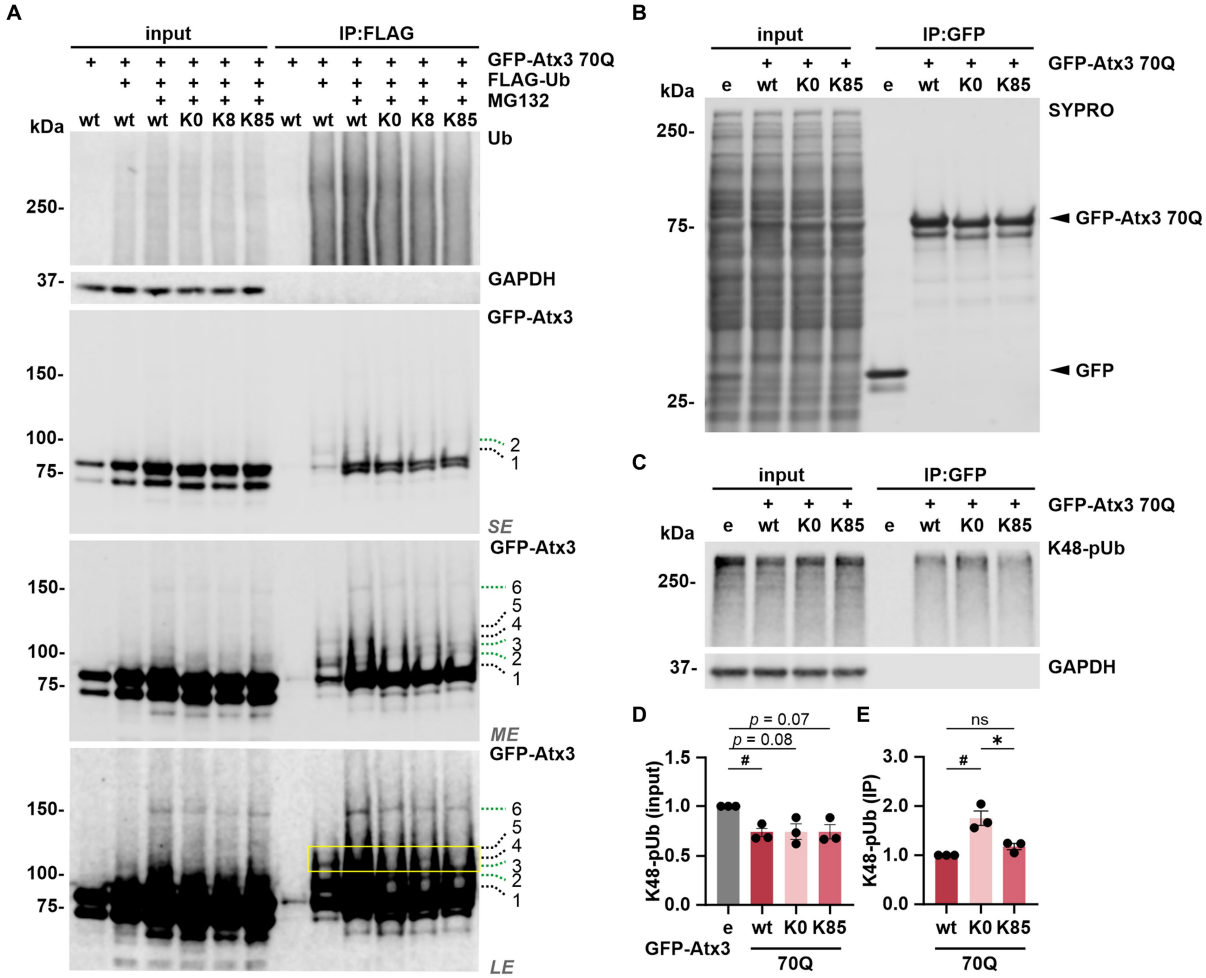
Lysine 8 is potentially ubiquitinated and lysine 85 regulates the ataxin-3 binding to K48-linked polyubiquitin chains. **(A)** Ubiquitination analysis of ataxin-3 by immunoprecipitation (IP) of FLAG-tagged ubiquitin. GFP-Atx3 and FLAG-Ub constructs were overexpressed in 293 T *ATXN3*^−/−^ cells, which were further treated with MG132 and subjected to FLAG tag-specific IP. Western blot analysis demonstrated that all GFP-Atx3 variants, including 70Q K0, were ubiquitin-positive, likely due to the presence of the GFP tag (green dashed lines 2, 3, and 6). Presence of specific, high-molecular weight ubiquitinated ataxin-3 was observed for 70Q wt (black dashed lines 1, 4, and 5), which was not the case for the 70Q K0 variant. Reintroduction of lysine residue K8 (70Q K8) restored one of the high molecular weight bands observed in 70Q wt (yellow box; black dashed line 4), suggestive of ubiquitination induced by the presence of K8. GAPDH served as loading and IP purity control. *SE*, short exposure; *ME*, medium exposure; *LE*, long exposure. *n* = 3. **(B–E)** Co-IP-based ataxin-3 interaction analysis. **(B)** Western blot of immunoprecipitated GFP empty (e) and different GFP-Atx3 70Q lysine variants from transfected 293 T *ATXN3*^−/−^ cells. IP-based purification was confirmed via SYPRO Ruby staining. **(C)** Western blot of the IP probed for co-precipitated K48-pUb chains. GAPDH served as loading and purity control. **(D,E)** Densitometric quantification of K48-pUb chains from **(C)**. **(D)** K48-pUb chains in the input were reduced upon the expression of Atx3 70Q wt in comparison to GFP empty, after normalization to GAPDH, whereas it showed a trend to reduction with Atx3 70Q K0 and 70Q K85. *n* = 3, one-sample *t*-test, empty (e) vs. 70Q wt, *p* = 0.03. **(E)** Immunoprecipitated GFP-Atx3 demonstrated increased binding to GFP-Atx3 70Q K0 in comparison to 70Q wt, after normalization to ataxin-3. Presence of lysine residue K85 (70Q K85) lowered the amount of bound K48-pUb chains back to 70Q wt levels. *n* = 3, one-sample *t*-test, 70Q wt vs. 70Q K0, *p* = 0.04; unpaired *t*-test. 70Q K0 vs. 70Q K85, *p* = 0.02. Data is represented as means ± SEM. *, ^#^ *p* ≤ 0.05.

Next, we sought to investigate whether ubiquitination, the primary PTM on lysine residues, occurs at these sites. For this, 293 T *ATXN3*^−/−^ cells were co-transfected with FLAG-Ub and either GFP-Atx3 70Q wt, 70Q K0, 70Q K8 or 70Q K85 for immunoprecipitation (IP) of ubiquitinated proteins via the FLAG tag. Cells transfected with 70Q wt only were used as controls. Western blot analysis of ubiquitin (Ub) and GFP demonstrated a successful IP of FLAG-Ub and ubiquitinated forms of GFP-Atx3 (Figure 5A). All GFP-Atx3 variants were ubiquitin-positive, likely due to ubiquitination of the GFP tag. Bands of ubiquitinated GFP-Atx3 were observed at around 100 kDa and above for Atx3 70Q wt (Figure 5A, indicated by dashed lines 1, 2, 3, 4, 5, and 6). Some of these bands were not present in Atx3 70Q K0 (black dashed lines 1, 4 and 5), whereas distinct high molecular weight bands were observed for all Atx3 70Q constructs (green dashed lines 2, 3 and 6). Moreover, one band was noticeably present upon reintroduction of lysine residue K8 (Figure 5A, black dashed line 4), which was not observed for Atx3 70Q K0 or K85. Therefore, we concluded that K8 in ataxin-3 is either a potential target site for ubiquitination, or its presence induced ubiquitination of ataxin-3 at a non-canonical site, since the lysine residue K8 is the only classical ubiquitination site available within the Atx3 70Q K8 variant.

To test whether ubiquitination occurs at K8, we performed mass spectrometry (MS)-based analysis of immunoprecipitated GFP-tagged ataxin-3 from cells treated with DMSO (GFP only and Atx3 70Q wt) or with the proteasome inhibitor MG132 (Atx3 70Q wt, 70Q K0, 70Q K8, and 70Q K85) for enriching ubiquitinated forms of ataxin-3. Our MS-based analysis of ataxin-3 variants validated previously described ubiquitinated lysine residues within ataxin-3 (Supplementary Figure S6; Supplementary Table S4; Todi et al., 2010). Moreover, it confirmed the suspected presence of ubiquitination in the GFP tag (Supplementary Table S4). However, peptides containing ubiquitinated K8 or K85 of ataxin-3 were not identified with this approach.

Finally, the absence of a marked ubiquitination on K85 suggests that this residue *per se* may contribute to the observed restoration of ataxin-3’s intracellular distribution (Figures 2B,C) via modulating the functionality of NES 77 (Figure 1A).

### K85 regulates the binding of ataxin-3 to K48-linked polyubiquitin chains

As lysine 85 is located between the ubiquitin-binding sites 1 and 2 (UbS1 and UbS2, respectively; Figure 1A) of ataxin-3, motifs that are relevant for the binding of polyubiquitin chains as a deubiquitinase (Nicastro et al., 2009) and for the protein stability by halting its proteasomal degradation (Blount et al., 2014), we analyzed the impact of lysine 85 on ataxin-3 binding to ubiquitin chains. We performed a GFP tag-directed co-IP using 293 T *ATXN3*^−/−^ cells overexpressing GFP alone or GFP-Atx3 70Q wt, 70Q K0 or 70Q K85 (Figure 5B). Western blot analysis of K48-pUb chains, which we reported to be greatly processed by ataxin-3 (Pereira Sena et al., 2021) via a mechanism widely dependent on the integrity of its UbS2 site (Nicastro et al., 2010), was generally reduced upon the presence of all ataxin-3 analyzed variants, although not reaching statistical significance with Atx3 70Q K0 or K85 (Figures 5C,D). Moreover, IP of GFP-Atx3 revealed a stronger binding of K48-pUb to Atx3 70Q K0 when compared to 70Q wt, whereas K85 normalized the affinity back to wild-type levels (Figures 5C,E). Based on these findings, we concluded that lysine 85 may be relevant for the regulation of ataxin-3’s UbS binding to K48-pUb chains. Noteworthily, we detected the binding of the highly conserved ATPase valosin-containing protein (VCP) (Zhong and Pittman, 2006) to Atx3 70Q K0 and K85 (Supplementary Figure S7), despite they carry a K283R mutation within the VCP-binding motif (VBM) of ataxin-3 (Boeddrich et al., 2006). Interestingly, mouse ataxin-3 also features an arginine at the 283 position (UniProt: Q9CVD2).

### K8/K85-double lysine ataxin-3 does not show a combinatorial effect of these residues

**Figure 6:**
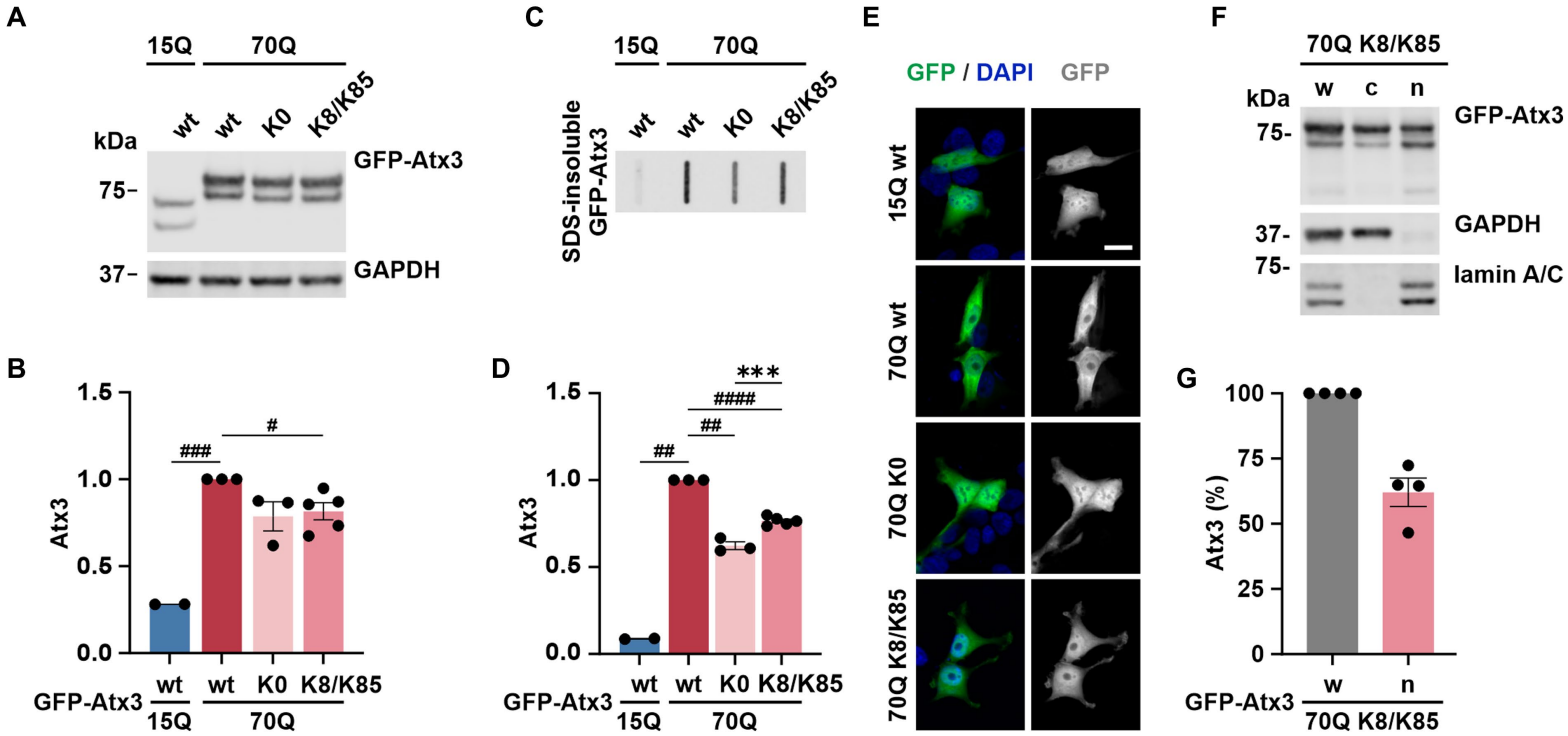
K8/K85 double mutant ataxin-3 show increased aggregation and nuclear localization. **(A)** Western blot analysis of protein extracts from 293 T *ATXN3*^−/−^ cells transfected with GFP-Atx3 variants. GAPDH served as loading control. **(B)** Densitometric quantification of soluble ataxin-3 from **(A)**. Comparable protein levels between Atx3 70Q K0 and 70Q K8/K85 double mutant were observed after normalization to GAPDH and relative to 70Q wt. *n* = 2–5, 70Q wt vs. 15Q wt, *p* = 0.0005, vs. 70Q K8/K85, *p* = 0.02 (#, one-sample *t*-test). **(C)** Filter retardation assay for analyzing SDS-insoluble forms of ataxin-3 in the same protein extracts. **(D)** Densitometric quantification of SDS-insoluble ataxin-3 relative to Atx3 70Q wt demonstrated an increased aggregate load of Atx3 70Q K8/K85 double mutant when compared to 70Q K0. *n* = 2–5, 70Q wt vs. 15Q wt, *p* = 0.001; vs. 70Q K0, *p* = 0.004; vs. 70Q K8/K85, *p* < 0.0001 (#, one-sample *t*-test); 70Q K0 vs. 70Q K8/K85, *p* = 0.0006 (*, unpaired *t*-test). **(E)** Fluorescence microscopy analysis demonstrated comparable intracellular distribution of Atx3 70Q K8/K85 and 70Q K0 in transfected 293 T *ATXN3*^−/−^ cells. Scale bar = 20 μm. **(F)** Western blotting of the subcellular fractionation of 293 T *ATXN3*^−/−^ cells overexpressing Atx3 70Q K8/K85 confirmed an enriched nuclear localization of this ataxin-3 variant. GAPDH and lamin A/C served as enrichment markers for the cytoplasmic and nuclear fractions (respectively), and as loading controls. w, whole cell extract; c, cytoplasmic fraction; *n*, nuclear fraction. **(G)** Densitometric quantification of the nuclear fraction of ataxin-3 from the experiments represented in **(F)**. Percentages were calculated relative to ataxin-3 in the whole cell extract, after normalization to nuclear protein lamin A/C. Data is represented as means ± SEM. *, ^#^ *p* ≤ 0.05, **, ^##^ *p* ≤ 0.01, ***, ^###^ *p* ≤ 0.001, ****, ^####^ *p* ≤ 0.0001.

As both lysine residues 8 and 85 demonstrated effects on ataxin-3 aggregation and intracellular distribution, we investigated whether such events would be intensified by the combined presence of both sites. For this, 293 T *ATXN3^−/−^* cells were transfected with GFP-Atx3 15Q wt, 70Q wt, 70Q K0 or 70Q containing both K8 and K85 residues (70Q K8/K85) and analyzed via western blotting (Figure 6A). The previously observed difference between Atx3 15Q wt and 70Q wt levels (Figure 1B) was reproducible. However, while soluble levels of Atx3 70Q K8 or K85 were comparable to 70Q wt (Figures 1B,C), Atx3 70Q K8/K85 demonstrated reduced protein levels, which was similar to 70Q K0 (Figures 6A,B).

Next, we analyzed SDS-insoluble forms of ataxin-3 using filter retardation assays. Analysis of the homogenates, aside from reproducing the previous findings on aggregation of Atx3 70Q wt and K0 (Figures 1D,E), demonstrated increased amounts of SDS-insoluble Atx3 70Q K8/K85 when compared to 70Q K0 (Figures 6C,D), however not reaching levels of 70Q wt. This result indicates an increased aggregation propensity of Atx3 70Q mediated by both lysine residues, whereas the elevated aggregation of single-lysine ataxin-3 70Q K8 or K85 appeared to directly result from the increased levels of the soluble protein (Figures 1B–E).

To evaluate potential changes in the intracellular distribution of Atx3 70Q K8/K85, we performed localization analysis using fluorescence microscopy. Interestingly, Atx3 70Q K8/K85 pointed to a slightly increased nuclear localization (Figure 6E), therefore comparable to 70Q K0 and 70Q K8 (Figure 2). To validate this finding, we performed a subcellular fractionation analysis under the same experimental conditions. Western blot analysis of the cellular fractions demonstrated that about 60% of Atx3 70Q K8/K85 was in the nucleus (Figures 6F,G), meaning a certainly strong nuclear presence of double-mutant ataxin-3.

Thus, Atx3 70Q K8/K85 does not directly combine the effects observed for Atx3 70Q K8 or K85 with regards to protein levels, but it shows an increased aggregate formation and enhanced intranuclear localization, pointing toward a potential pathophysiological role of both lysine residues in polyQ-expanded ataxin-3 toxicity.

## Discussion

Lysine residues are one of the main sites for PTMs in a protein and their modification can influence, among other functions, protein stability, activity, and subcellular localization (Wang and Cole, 2020). Therefore, we analyzed the relevance of lysine residues in the molecular characteristics of polyQ-expanded ataxin-3, a DUB that is altered in MJD. In our study, we demonstrated that lysine-free ataxin-3 is present at lower protein levels, while lysine residues K8 and K85, located in the catalytic Josephin domain, were essential for maintaining soluble and SDS-insoluble levels of ataxin-3. Further, we showed that K85, which is located within NES 77 (I77-L89) (Antony et al., 2009), contributes to the nucleocytoplasmic shuttling of ataxin-3 by lowering its nuclear localization. Next, we provided evidence that both K8 and K85 residues had stabilizing effects on the ataxin-3 protein turnover. All ataxin-3 variants were predominantly degraded via the UPS, and preventing this degradation increased cell death via apoptosis in cells expressing Atx3 70Q K0, whereas reintroduction of K8 or K85 improved cell survival. We detected a likely ubiquitination on K8, whereas K85 demonstrated to be relevant for the K48-linked pUb chain binding of ataxin-3, as the presence of this specific lysine residue reversed the elevated binding capability observed for the DUB’s lysine-free variant. Moreover, Atx3 70Q K8/K85 double mutant did not combine the effects on protein aggregation observed for the single residues K8 and K85, but the relatively increased aggregation propensity compared to 70Q K0, and the strong nuclear presence when compared to other ataxin-3 variants, suggest that both sites might influence the pathogenicity of polyQ-expanded ataxin-3. Finally, we demonstrated that lysine-free and single-lysine ataxin-3 K8 or K85 reproduced various physiological characteristics of unmutated polyQ-expanded ataxin-3, including protein localization, degradation, aggregation, ubiquitin-binding function, and protein–protein interaction.

In our experiments, proteasomal degradation was the main modifier of ataxin-3 aggregation, since inhibition of the UPS promoted a further accumulation of ataxin-3 aggregates in comparison to autophagy inhibition. As we also observed a stronger accumulation of ataxin-3 soluble levels with proteasome inhibition, we concluded that the increased levels of ataxin-3 aggregates directly reflected the total protein amount of ataxin-3. Our results correspond to earlier findings on proteasome inhibition in lactacystin-treated cells expressing GFP-tagged huntingtin exon 1 (Wyttenbach et al., 2000; Ravikumar et al., 2002). On the other hand, the detected effects on aggregation may still occur disconnected from repercussions on the soluble ataxin-3 protein, as proteasomes were shown to degrade intranuclear inclusion bodies directly (Iwata et al., 2009). In stark contrast to this, proteasome-dependent formation of polyQ peptides was associated with aggregate initiation and increased neurotoxicity (Raspe et al., 2009). Lysine-free ataxin-3 had a strongly reduced protein level in its non-pathogenic form (15Q K0), whereas pathologically expanded ataxin-3 without lysine residues (70Q K0) presented a trend toward reduced protein levels (in comparison to Atx3 15Q and 70Q, respectively), which translated into a highly significant lower aggregate load. Lysine-free ataxin-3 had been already reported to be less stable than its wild-type counterpart (Blount et al., 2014), where the authors propose that its degradation is through the interaction between the ubiquitin-binding site 2 (UbS2) with Rad23. In our CHX chase experiments, presence of the residues K8 or K85, the latter located between UbS1 and UbS2, led to increased ataxin-3 protein stability. Therefore, we conclude that the UbS-adjacent K85 as well as K8 contribute to the modulation of ataxin-3 turnover. Furthermore, both residues improved cell viability, compared to the detrimental Atx3 70Q K0 variant, with an overcompensation upon 70Q K85 expression. This effect might be attributed to the recovery of an active NES 77 due to K85 reactivation, thus rescuing the physiological cellular distribution of ataxin-3. Detectable changes in the ubiquitination of ataxin-3, however, were not induced by this lysine residue.

On the other hand, with Atx3 70Q K8 we observed alterations in the ubiquitin-linked band pattern of ataxin-3, indicating a potential ubiquitination at this site. This is in line with previous findings reported in a mass spectrometry-based study of ataxin-3 (Kristensen et al., 2017). Although we cannot conclude that the increased stability of Atx3 70Q K8 is related to its potential ubiquitination induced by K8, this earlier reported PTM might cause the formation of enlarged ataxin-3 aggregates, herein detected as an increased aggregate load. Comparable associations were made in the context of Huntington’s disease, where ubiquitination of K6 and K9 within huntingtin led to the formation of fewer but larger protein aggregates (Hakim-Eshed et al., 2020). Likewise, our experiments with Atx3 70Q K8/K85 strengthened the contribution of these sites to ataxin-3 aggregation. Future analyses with K8R/K85R mutations in ataxin-3 might help to clarify the exact role of these residues on the protein’s propensity to aggregate.

The catalytic activity of ataxin-3 toward K48-linked pUb chains as well as K48-K63-mixed chains depends on UbS2, and mutating the amino acid tryptophan at position 87 to an alanine reportedly impaired the ability of ataxin-3 to cleave K48-linked chains (Nicastro et al., 2010). Here we demonstrated that Atx3 70Q K0 bound more K48-linked polyubiquitin chains in comparison to 70Q wt, whereas the presence of K85, which is located between UbS1 and UbS2, partly restored its binding. This observation might suggest that the lysine-free variant instead has a normal binding (via its intact UIMs) but an insufficient proteolytic processing of K48-linked pUb chains, impairing the release of unprocessed substrate. K85 thus likely restored the UbS2 function of Atx3 70Q K85, which would explain the normal levels of K48-linked pUb chains bound to this ataxin-3 variant. These results highlight the importance of lysine residue K85 for ataxin-3’s affinity and processing of K48-linked pUb chains through the previously reported joint action of its UIMs and UbS2 (Blount et al., 2014).

K85 is also located inside of one of the two known nuclear export signals of ataxin-3 (Antony et al., 2009). Interestingly, lysine-free ataxin-3 localized more in the nucleus, and the presence of K85 restored its subcellular distribution, suggesting that K85 is decisive for the cytoplasmic localization of ataxin-3. However, the mere concomitant presence of K8 was sufficient to halt this effect. Whether the occurrence of a PTM at K8 or K85 contributes to ataxin-3’s subcellular localization remains unclear, but masking of localization signals, either by PTMs, protein–protein interactions or intrinsic conformational changes has been reported for other proteins (Chen and Mallampalli, 2009; McLane and Corbett, 2009).

Although lysine residues are generally known as the primary site for ubiquitination, a total of 15 different PTM types are known to target these sites - the largest number among all amino acids (Ramazi and Zahiri, 2021). Hence, it cannot be ruled out that alternative PTMs occur on the investigated sites K8 and K85, which may explain the observed effects on ataxin-3 when eliminating or reintroducing the respective residues.

Our results highlight the unequivocal impact of single lysine residues K8 and K85 within ataxin-3 on the polyQ-expanded protein’s pathophysiology. They corroborate the importance of these target sites for PTMs, underline their potential role in modulating MJD pathogenesis, and inspire future research on the nature of occurring modifications.

## Data availability statement

The original contributions presented in the study are included in the article/Supplementary material, further inquiries can be directed to the corresponding author.

## Author contributions

PS, JW, HN, OR, and TS conceptualized the study. PS, JW, SB, RS, RE, XL, AV, and JJ conducted the experiments. PS, JW, AV, and BM analyzed and interpreted the data. PS prepared the figures. PS and JW wrote the initial version of the manuscript. All authors proofread and agreed to the published version of the manuscript.

## Funding

PS received funding from the Brazilian National Council for Scientific and Technological Development (CNPq) and the German Academic Exchange Service (DAAD) within the “Science without Borders” program (process no. 229957/2013-7). JW received funding from the German Research Foundation (DFG; research grant no. WE 6585/1-1). XL received funding from the Natural Science Foundation of Hebei Province (C2021203004). HN received funding from the German Research Foundation (DFG; research grant no. NG 101/6-1). This study was supported by the German Federal Ministry of Education and Research (BMBF, E-Rare project SCA-CYP, grant no. 01GM1803; EuSAge project, grant no. 01DN18020).

## Conflict of interest

The authors declare that the research was conducted in the absence of any commercial or financial relationships that could be construed as a potential conflict of interest.

## Publisher’s note

All claims expressed in this article are solely those of the authors and do not necessarily represent those of their affiliated organizations, or those of the publisher, the editors and the reviewers. Any product that may be evaluated in this article, or claim that may be made by its manufacturer, is not guaranteed or endorsed by the publisher.

## Footnotes

^1^ https://primer3.ut.ee/
