# Supplementary material for: Implications of specific lysine residues within ataxin-3 for the molecular pathogenesis of Machado-Joseph disease
Source: Front Mol Neurosci. 2023 May 19;16:1133271. doi: 10.3389/fnmol.2023.1133271 (PMC10235640; doi:10.3389/fnmol.2023.1133271)
Supplement: Supplementary file 1 [file Data_Sheet_1.pdf]

Frontiers in Molecular Neuroscience

Original Research

Implications of specific lysine residues of ataxin-3 for the molecular pathogenesis of Machado-Joseph disease

Priscila Pereira Sena[1], Jonasz Jeremiasz Weber[1,2], Sercan Bayezit[1], Rafael Saup[1], Rana Dilara Incebacak Eltemur[1,2], Xiaoling Li[1], Ana Velic[3], Jaqueline Jung[1], Boris Macek[3], Huu Phuc Nguyen[2], Olaf Riess[1], Thorsten Schmidt[1,*]

[1] Institute of Medical Genetics and Applied Genomics, University of Tübingen, Tübingen, Germany

[2] Department of Human Genetics, Ruhr University Bochum, Bochum, Germany

[3] Proteome Center Tübingen, University of Tübingen, 72076, Tübingen, Germany

*** Correspondence**

Thorsten Schmidt

thorsten.schmidt@med.uni-tuebingen.de

Supplementary data

Supplementary figures

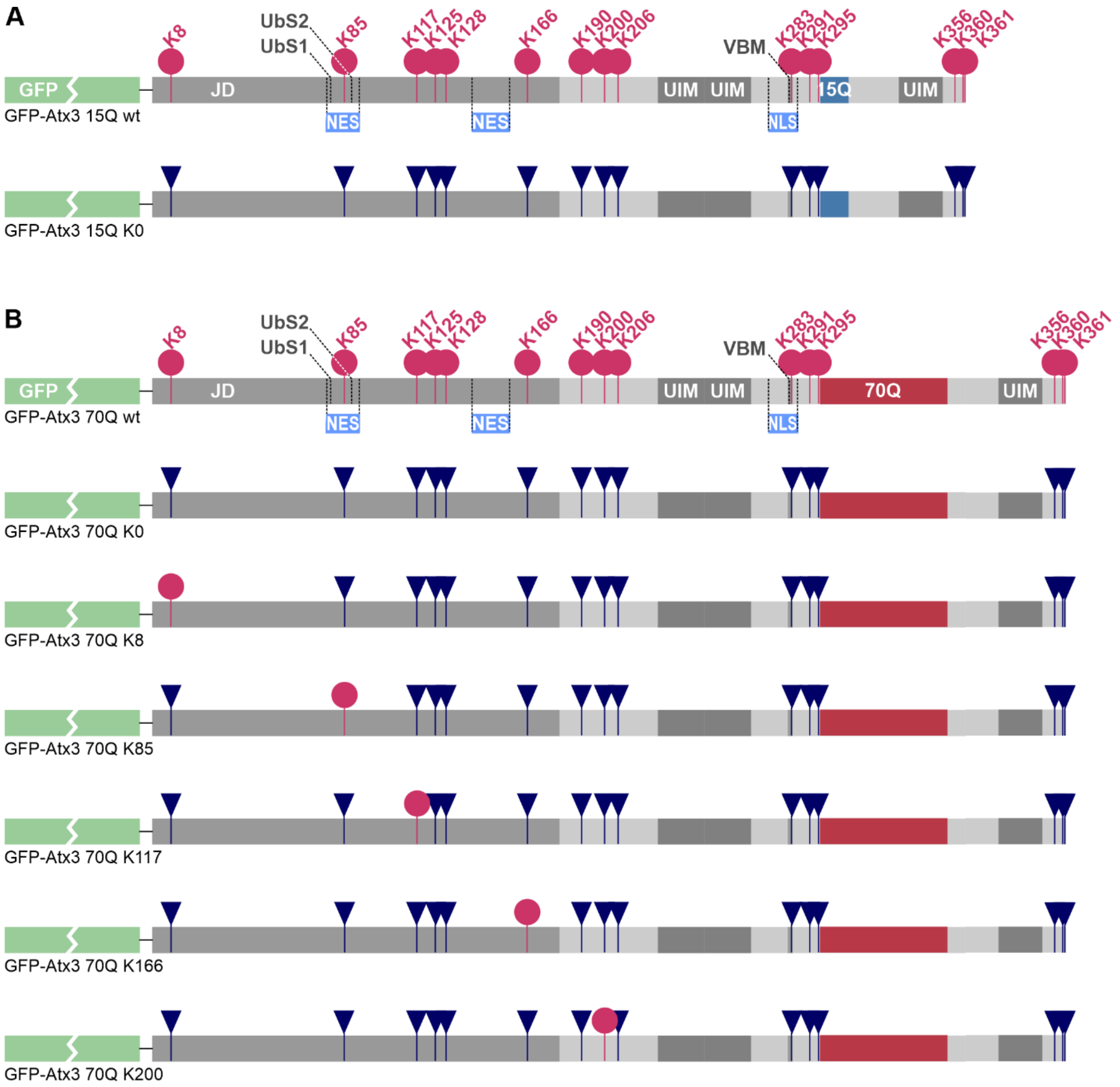

Supplementary Figure 1. Schematic representation of the GFP-tagged ataxin-3 overexpression constructs used in this study. All lysine residues (K; pink circles) within ataxin-3 containing 15 **(A)** or 70 **(B)** glutamines (Q) were converted to arginine (R; dark blue inverted triangles). **(B)** Single lysine residues of interest within Atx3 70Q were reactivated (exemplified by the pink circles). JD, Josephin domain; NES, nuclear export signal; NLS, nuclear localization signal; pQ, polyglutamine stretch; UbS, ubiquitin-binding site; UIM, ubiquitin-interacting motif; VBM, VCP-binding motif.

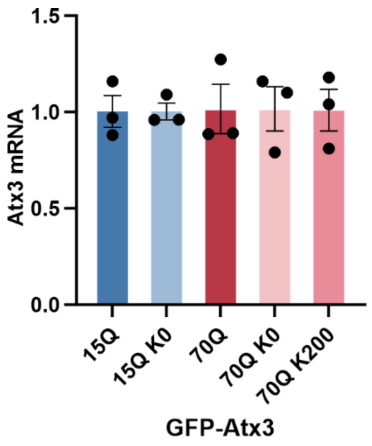

Supplementary Figure 2. Ataxin-3 variants do not differ in their mRNA expression levels. Analysis via qRT-PCR for GFP-Atx3 expression in respectively transfected 293T *ATXN3*$^{-/-}$ cells presented no differences at the mRNA level between the investigated ataxin-3 variants, with neither CAG repeat length nor presence or absence of lysine residues showing effects.

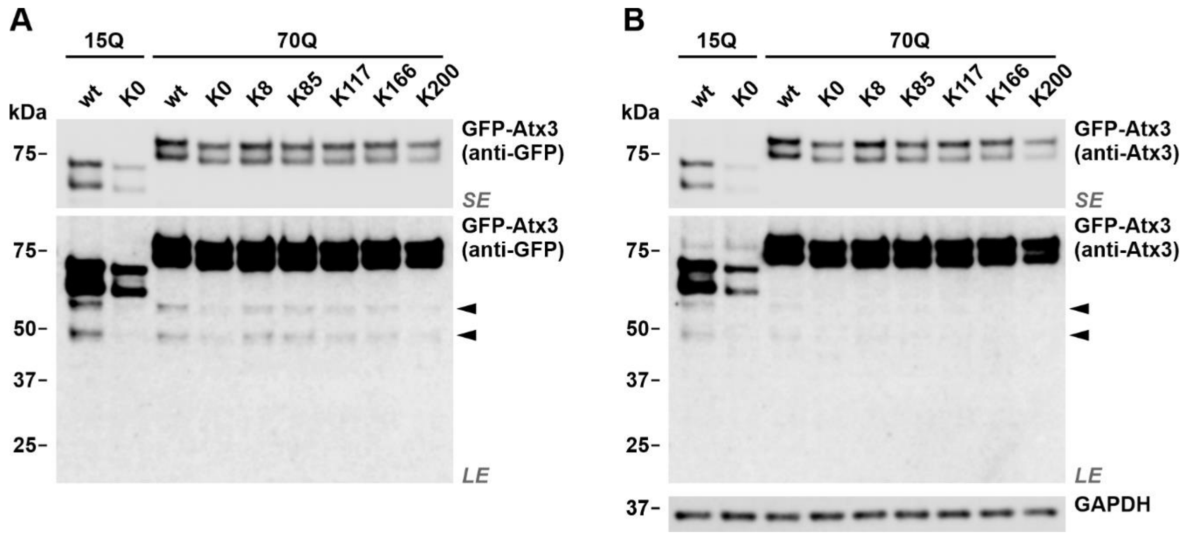

Supplementary Figure 3. Ataxin-3 variants do not show major changes in their baseline fragmentation pattern. (A and B) Western blot analysis of protein extracts from 293T *ATXN3*$^{-/-}$ cells transfected with various GFP-tagged ataxin-3 (GFP-Atx3) variants for 72 h. The same membrane was probed with antibodies against GFP **(A)** or the N-terminus of ataxin-3 **(B)**. GAPDH served as loading control. Arrowheads point to potential proteolytic fragments of ataxin-3. *SE* = short exposure, *LE* = long exposure.

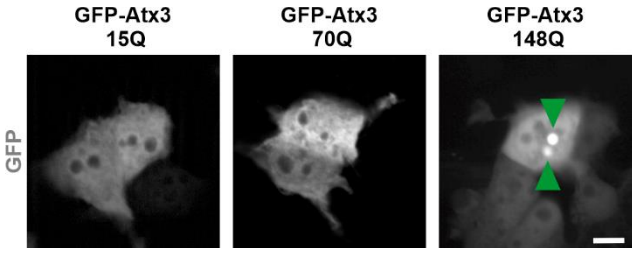

Supplementary Figure 4. Ataxin-3 70Q does not form microscopically detectable intracellular inclusion bodies. Fluorescence microscopy analysis of 293T *ATXN3*$^{-/-}$ cells transfected with GFP-Atx3 15Q, 70Q, or 148Q for 72 h demonstrated a comparable intracellular distribution of ataxin-3 independent of its polyQ length. All ataxin-3 variants are evenly localized in the cytoplasm and the nucleus, while only GFP-Atx3 148Q formed microscopically visible intracellular inclusion bodies (green arrowheads). Scale bar = 10 µm.

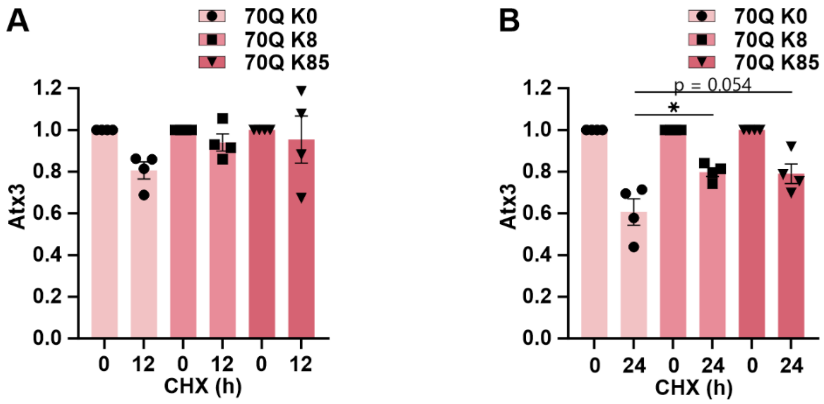

Supplementary Figure 5. Stability of ataxin-3 is increased by reactivation of K8 and K85. Densitometric quantification of soluble protein levels of ataxin-3 at the 12-h **(A)** or 24-h **(B)** time points as assessed by the cycloheximide (CHX) chase assay (see Figure 3A). n = 4, one-way ANOVA with Tukey post-hoc test, 70Q K0 vs 70Q K8, $p = 0.04$.

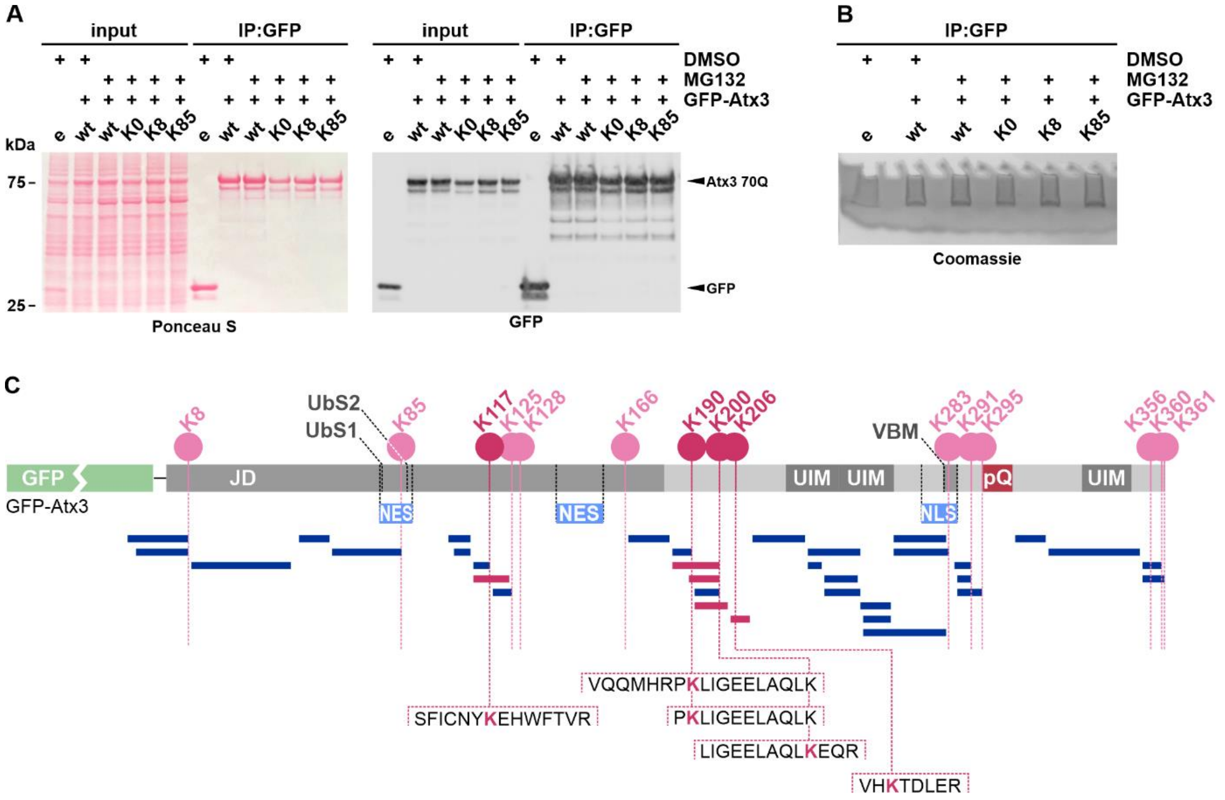

Supplementary figure 6. Identification of ubiquitinated lysine residues within ataxin-3 via mass-spectrometry analysis. (A) Western blotting of immunoprecipitated (IP) GFP-tagged ataxin-3 (GFP-Atx3). GFP-Atx3 constructs were overexpressed in 293T *ATXN3*$^{-/-}$ cells, which were further treated with DMSO or MG132 and subjected to GFP tag-specific IP. Ponceau S staining served as loading and purity control and the membrane was probed to GFP. **(B)** Samples from (A) were subjected to a short electrophoresis before a Coomassie brilliant blue gel staining, followed by the excision of the sample-containing lanes for mass spectrometry (MS) analysis. **(C)** Graphical representation of the resulting MS-based analysis of ubiquitination in ataxin-3. Horizontal bars represent the identified peptides. Blue bars, unmodified peptides. Red bars, peptides containing ubiquitinated lysine residues (GlyGly modified). Specific amino acid sequences of the peptides containing ubiquitinated sites (K) are also represented. Lysine residues in pink represent not covered or not ubiquitinated sites.

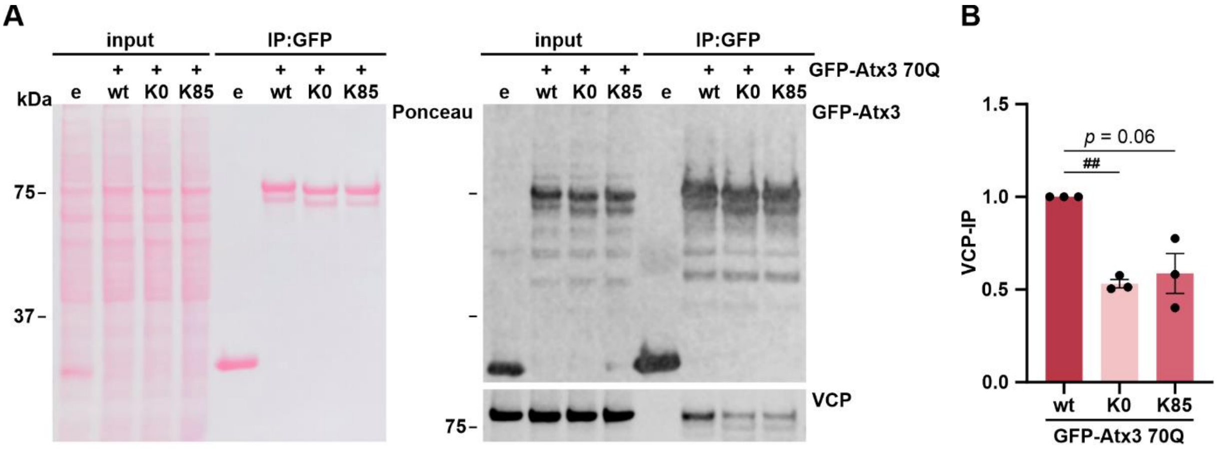

Supplementary Figure 7. Lysine-free and single lysine ataxin-3 conserve the physiological binding to the ATPase valosin-containing protein. (A) Co-IP-based ataxin-3 interaction analysis. Western blot of immunoprecipitated GFP empty (e) and different GFP-Atx3 70Q lysine variants expressed in 293T *ATXN3*$^{-/-}$ cells. IP-based purification was confirmed via Ponceau S staining. Membrane was probed for the co-precipitated ataxin-3 interaction partner ATPase valosin-containing protein (VCP). **(B)** Densitometric quantification of VCP from (A) demonstrated a conserved, yet reduced binding to GFP-Atx3 70Q K0 and 70Q K85 in comparison to 70Q wt. n = 3, one-sample *t*-test, 70Q wt vs 70Q K0, $p = 0.002$. Data is represented as means ± SEM. $^{\#\#}$ $p \leq 0.01$.

Supplementary Tables

Supplementary Table 1. Nucleotide sequences of gBlocks™ used for cloning lysine-free and single lysine-reactivated GFP-ataxin-3 constructs. Nucleotides underlined indicate the cutting site for the restriction enzymes listed in Supplementary Table 2.

**gBlock A:**<br>ccggactcagatctcgagctccttaagcccaggacctggcatggagtccatcttccacgag<br>aaacaagaaggctcactttgtgctcaacattgcctgaataacttattgcaaggagaatatt<br>ttagccctgtggaattatcctcaattgcacatcagctggatgaggaggagaggatgagaat<br>ggcagaaggaggagttactagtgaagattatcgcacgtttttacagcagccttctggaaat<br>atggatgacagtggtttttctctattcaggttataagcaatgccttgaaagtttggggtt<br>tagaactaatcctgttcaacagtccagagtatcagaggctcaggatcgatcctataaatga<br>aagatcatttatatgcaattataaggaacactggtttacagttagaaaattaggaaaacag<br>tggtttaacttgaattctctcttgacgggtccagaattaatatcagatacatatcttgcac<br>ttttcttggctcaattacaacaggaaggttattctatatttgtcgttaagggtgatctgcc<br>agattgcgaagctgaccaactcctgcagatgattagggtccaacagatgcatcgaccaaaa<br>cttattggagaagaattagcacaactaaaagagcaaagagtccataaaacagacctggaac<br>gagtgttagaagcaaatgatggctcaggaatgttagacgaagatgaggaggatttgcagag<br>ggctctggcactaagtcgccaagaaattgacatggaagatgaggaagcagatctccgcagg<br>gctattcagctaagtatgcaaggtagttccagaaacatatctcaagatatgacacagacat<br>caggtacaaatcttacttcagaagagcttcggaagagacgagaagcctactttgaaaaaca<br>gcagcaaaagcagcaacagcaacagcaacagcaacagcaacagcaacagcaacaggggac<br>ctatcaggacagag
**gBlock B:**<br>ccggactcagatctcgagctccttaagcccaggacctggcatggagtccatcttccacgag<br>aaacaagaaggctcactttgtgctcaacattgcctgaataacttattgcaaggagaatatt<br>ttagccctgtggaattatcctcaattgcacatcagctggatgaggaggagaggatgagaat<br>ggcagaaggaggagttactagtgaagattatcgcacgtttttacagcagccttctggaaat<br>atggatgacagtggtttttctctattcaggttataagcaatgccttgaaagtttggggtt<br>tagaactaatcctgttcaacagtccagagtatcagaggctcaggatcgatcctataaatga<br>aagatcatttatatgcaattatagggaacactggtttacagttagaagattaggaagacag<br>tggtttaacttgaattctctcttgacgggtccagaattaatatcagatacatatcttgcac<br>ttttcttggctcaattacaacaggaaggttattctatatttgtcgttaagggtgatctgcc<br>agattgcgaagctgaccaactcctgcagatgattagggtccaacagatgcatcgaccaaga<br>cttattggagaagaattagcacaactaaaagagcaaagagtccatagaacagacctggaac<br>gagtgttagaagcaaatgatggctcaggaatgttagacgaagatgaggaggatttgcagag<br>ggctctggcactaagtcgccaagaaattgacatggaagatgaggaagcagatctccgcagg<br>gctattcagctaagtatgcaaggtagttccagaaacatatctcaagatatgacacagacat<br>caggtacaaatcttacttcagaagagcttcggaagagacgagaagcctactttgaaagaca<br>gcagcaaaagcagcaacagcaacagcaacagcaacagcaacagcaacagcaacaggggac<br>ctatcaggacagag

Supplementary Table 2. Nucleotide sequences and restriction enzymes employed for generating lysine-free and single lysine-reactivated GFP-ataxin-3 constructs. All constructs are based on a pEGFP-C2 backbone carrying the cDNA of the canonical isoform of Atx3 (isoform 2, UniProt: P54252-2). **R**, A or G; **W**, A or T; **Y**, C or T; **N**, A, T, C or G.

**EGFP-ataxin-3 construct**	**Restriction enzymes (including sites) or primers (R: reverse)**
K0 15Q/70Q<br>(lysine-free)	5’: SacI (5’ GAGCTC 3’)<br>3’: Esp3I (5’ GAGACG 3’)<br>3’: PpuMI (5’ RGGWCCY 3’)
K8 15Q/70Q<br>(reintroduced lysine 8)	5’: SacI (5’ GAGCTC 3’)<br>3’: SpeI (5’ ACTAGT 3’)
K85 15Q/70Q<br>(reintroduced lysine 85)	5’: SpeI (5’ ACTAGT 3’)<br>3’: EcoRI (5’ GAATTC 3’)
K117 15Q/70Q<br>(reintroduced lysine 117)	R:<br>gagagaattcaagttaaaccactgtcttcctaatcttctaactgtaaaccagtgttccttataattg
K166 15Q/70Q<br>(reintroduced lysine 166)	5’: EcoRI (5’ GAATTC 3’)<br>3’: PstI (5’ CTGCAG 3’)
K200 15Q/70Q<br>(reintroduced lysine 200)	5’: PstI (5’ CTGCAG 3’)<br>3’: XmnI (5’ GAANNNNTTC 3’)

Supplementary Table 3. Primary antibodies for western blot analysis.

Antibody/target	Host	Dilution	Clone/catalog no.	Vendor
β-actin	mouse	1:5,000	AC-15/A5441	Sigma-Aldrich
ataxin-3	mouse	1:5,000	1H9/MAB5360	Merck
ataxin-3 (N-terminal)	rabbit	1:500	ARP50507_P050	Aviva
caspase-7	rabbit	1:1,000	#9492	Cell Signaling
GAPDH	mouse	1:2,000	0411/sc-47724	Santa Cruz
GFP	mouse	1:1,000	sc-9996	Santa Cruz
LC3B	rabbit	1:1,000	#2775	Cell Signaling
PARP1	rabbit	1:2,500	13371-1-AP	ProteinTech
ubiquitin	mouse	1:1,000	P4D1/sc-8017	Santa Cruz
ubiquitin (K48-linkage specific)	rabbit	1:1,000	D9D5/#8081	Cell Signaling
VCP	rabbit	1:250	sc-20799	Santa Cruz

Supplementary table 4. GFP-ataxin-3 peptides containing ubiquitinated (GlyGly) lysine (K) residues identified via mass spectrometry-based analysis.

**GlyGly (K) peptides**	**Protein**	**Sample**					
		GFP DMSO	70Q wt DMSO	70Q wt MG132	70Q K0 MG132	70Q K8 MG132	70Q K85 MG132
TIFF**K**DDGNYK	GFP		✓	✓	✓	✓	✓
LEYNYNSHNVYIMAD**K**QK	GFP		✓	✓			
SFICNY**K**EHWFTVR	Atx3		✓				
VQQMHRP**K**LIGEELAQLK	Atx3		✓	✓			
P**K**LIGEELAQLK	Atx3		✓	✓			
LIGEELAQL**K**EQR	Atx3		✓	✓			
VH**K**TDLER	Atx3		✓	✓			
